# A Pharmacokinetic Study of Sixteen Major Bioactive Components of Jinshui-Huanxian Granules in Pulmonary Fibrosis Model and Control Rats Using Orbitrap Fusion Mass Spectrometry

**DOI:** 10.3390/molecules28186492

**Published:** 2023-09-07

**Authors:** Weiwei Zhang, Yan Wan, Shuding Sun, Yang Xie, Di Zhao, Bing Li, Jiansheng Li, Yange Tian, Suxiang Feng

**Affiliations:** 1 Academy of Chinese Medical Sciences, Henan University of Chinese Medicine, Zhengzhou 450003, China; weiwei.zhang@yahoo.com (W.Z.); sunshuding@126.com (S.S.); zhaodiabcd@sina.com (D.Z.); 18838193637@163.com (B.L.); 2 Faculty of Chemistry, University of Strasbourg, 67008 Strasbourg, France; 3 College of Pharmacy, Henan University of Chinese Medicine, Zhengzhou 450003, China; wanyan1536@163.com; 4 Collaborative Innovation Center for Chinese Medicine and Respiratory Diseases Co-Constructed by Henan Province & Education Ministry of P. R. China, Zhengzhou 450046, China; xieyanghn@163.com (Y.X.); li_js8@163.com (J.L.); 5 The First Affiliated Hospital, Henan University of Chinese Medicine, Zhengzhou 450003, China

**Keywords:** Jinshui-Huanxian granules (JHGs), pharmacokinetic, pulmonary fibrosis (PF), bioactive compounds, Orbitrap Fusion MS

## Abstract

Jinshui-Huanxian granules (JHGs), a Chinese herbal compound prescription, have shown a therapeutic effect in reducing lung tissue damage, improving the degree of pulmonary fibrosis, replenishing lungs and kidneys, relieving cough and asthma, reducing phlegm, and activating blood circulation. However, these active compounds’ pharmacokinetics and metabolic processes were unclear. This study aimed to compare the pharmacokinetics, reveal the metabolic dynamic changes, and obtain the basic pharmacokinetic parameters of 16 main bioactive compounds after intragastric administration of JHGs in control and pulmonary fibrosis (PF) model rats by using Orbitrap Fusion MS. After administration of JHGs, the rat plasma was collected at different times. Pretreating the plasma sample with methanol and internal standard (IS) solution carbamazepine (CBZ), and it was then applied to a C_18_ column by setting gradient elution with a mobile phase consisting of methanol 0.1% formic acid aqueous solution. Detection was performed on an electrospray ionization source (ESI), and the scanning mode was SIM. Pharmacokinetic parameters were analyzed according to the different analytes’ concentrations in plasma. The matrix effect was within the range of 79.01–110.90%, the extraction recovery rate was 80.37–102.72%, the intra-day and inter-day precision relative standard deviation (RSD) was less than 7.76%, and the stability was good, which met the requirements of biological sample testing. The method was validated (r ≥ 0.9955) and applied to compare the pharmacokinetic profiles of the control group and PF model group after intragastric administration of the JHGs. The 16 analytes exhibited different pharmacokinetic behaviors in vivo. In the pathological state of the PF model, most of the components were more favorable for metabolism and absorption, and it was more meaningful to study the pharmacokinetics. Above all, this study provided an essential reference for exploring the mechanism of action of JHGs and guided clinical medication as well.

## 1. Introduction

Chinese Medicine (CM) has good efficacy in the treatment of pulmonary fibrosis (PF), which is pathologically characterized by an irreversible loss of lung function and structural changes with initial alveolitis predominating. It can be caused by secondary factors and idiopathic pulmonary fibrosis (IPF). As the disease progresses, it can cause inflammatory cell infiltration, collagen fiber proliferation, stromal deposition, and ultimately lead to irreversible scarring fibrosis of the alveolar wall, pulmonary vasculature, and also the airways [1]. The disease is also one of the most difficult diseases listed by the World Health Organization (WHO) [2]. The main clinical manifestations are dyspnea, chest tightness, irritating dry cough, low sputum, and some patients show wasting, loss of appetite, weakness, and cyanosis. Most patients die of respiratory failure, which is a serious threat to the quality of life and the health of human life [3,4]. According to European statistics, the incidence of PF ranges from 1.25 to 23.4/100,000, with a median survival of 2–3 years and a 5-year survival rate of less than 30% [5,6]. PF is characterized by a yearly increase in incidence, rapid progression, poor prognosis, short survival, and high rates of disability and death. There has been no major clinical breakthrough in treatments in Western medicine, in which it is currently treated mainly by anti-inflammatory, immunosuppressives, and lung transplantation, but the disadvantages are limited efficacy, high cost, and obvious side effects [7]. JHGs have shown clear efficacy in the treatment of pulmonary fibrosis [8]. They consists of 10 Chinese medicines, including *Epimedium brevicornu* Maxim, *Panax ginseng* C.A.Mey., *Ophiopogon japonicus* (Thunb.) Ker Gawl., Rehmannia glutinosa (Gaertn.) DC. Trichosanthes kirilowii Maxim., Fritillaria thunbergii Miq., *Ginkgo biloba* L., Citrus × aurantium f. deliciosa (Ten.) M.Hiroe, *Glycyrrhiza glabra* L., and *Paeonia officinalis* L., which have the effects of tonifying the lungs and kidneys, relieving cough and asthma, resolving phlegm, and activating blood. (All the herbs’ names could be found in “The Plant List” (www.theplantlist.org, (accessed on 23 August 2023)) or MPNS (https://mpns.science.kew.org), (accessed on 23 August 2023)). The clinical treatment evaluated the efficacy and long-term effects of JHGs in the treatment of PF from several indexes, with the effects of improving lung function, inhibiting inflammation, reducing lung tissue damage, and improving the degree of pulmonary fibrosis [9,10]. The efficacy and safety research of CM including JHG treatment for IPF to ensure the adverse reactions of JHGs were few and well-tolerated [8,11].

Furthermore, pharmacological studies found that JHGs had significant therapeutic and long-term effects on pulmonary fibrosis in rats by repairing the balance of Nrf_2_-NOX_4_ and decreasing the oxidative response [12]. The identification of effective compounds of JHGs could ameliorate fibroblast activation in PF by inhibiting the activation of mTOR signaling. JHGs’ potential pharmacological mechanisms were explored in IPF therapy using network intersection analysis and demonstrated seventy-two JHG targets were closely related to IPF, which could alleviate the degree of PF, including decreases in collagen deposition and epithelial–mesenchymal transition. Moreover, JHGs might suppress fibroblast activation by inhibiting the EGFR/PI_3_K/AKT signaling pathway to ameliorate PF. Tangeretin, isosinensetin, and Peimine might be the active compounds in JHGs that are involved in the treatment and that have therapeutic effects on IPF [9]. The complexity of JHGs’ ingredients and underlying mechanisms make us continuously identify its chemical compounds and dosage. The effective components have been screened by network pharmacology and molecule docking, and their underlying mechanisms for PF treatment have been clarified [9]. The components and molecular mechanisms of JHGs were characterized and integrated with network pharmacology. As a result, 266 components were identified in JHGs. A total of 37 components in JHGs were finally established based on ultra-high-performance liquid chromatography coupled with Orbitrap Fusion mass spectrometry (MS), providing a scientific basis for the quality evaluation and control of JHGs [10,13]. However, pharmacokinetics and the metabolic processes of these active compounds still deserve to be elucidated by more animal experiments. In particular, the comparative study between pathological and control states was always unclear.

**Figure 1:**
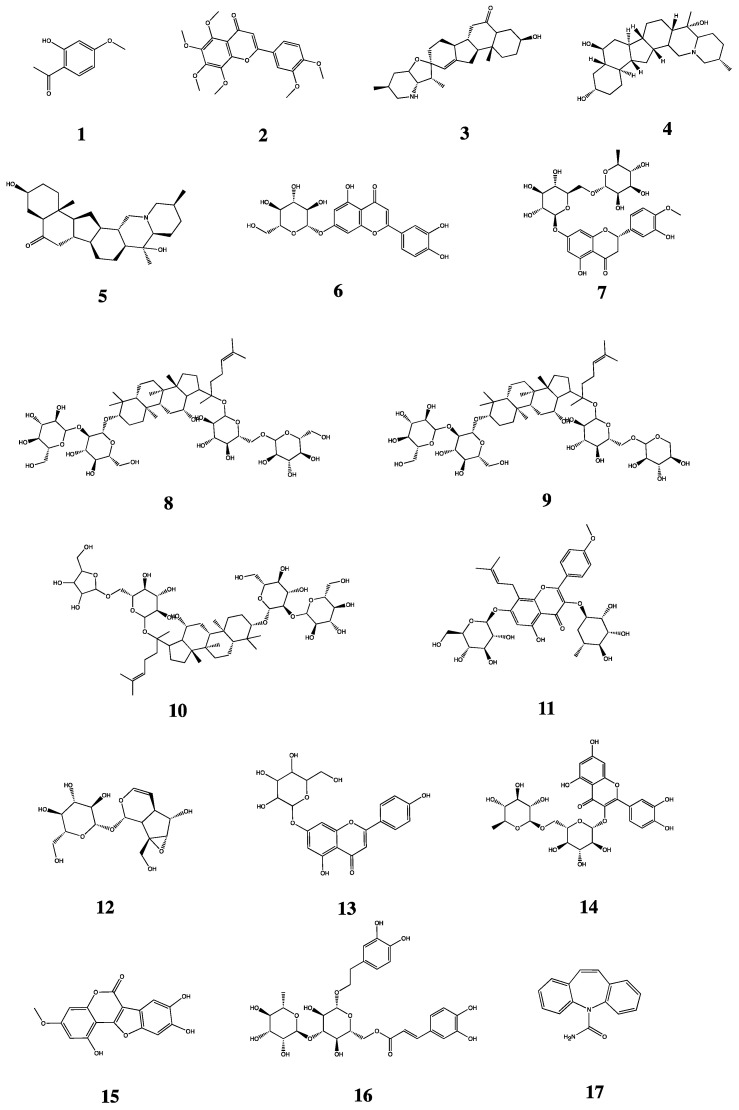
Chemical structures of (**1**) Paeonol, (**2**) Nobiletin, (**3**) Peimisine, (**4**) Peimine, (**5**) Peiminine, (**6**) Cynaroside, (**7**) Hesperidin, (**8**) Ginsenoside Rb_1_, (**9**) Ginsenoside Rb_2_, (**10**) Ginsenoside Rc, (**11**) Icariin, (**12**) Catalpol, (**13**) Apigenin-7-glucoside, (**14**) Rutin, (**15**) Wedelolactone, (**16**) Isoacteoside as analytes, and (**17**) Carbamazepine (CBZ) as internal standard (IS).

In our preliminary experiment, based on drug-active ingredients and serum chemistry studies, sixteen major bioactive components of JHGs were finally selected to obtain a pharmacokinetic study in the PF model and control rats, which were Paeonol, Nobiletin, Peimisine, Peiminine, Peimine, Cynaroside, Hesperidin, Ginsenoside Rb_1_, Ginsenoside Rb_2_, Ginsenoside Rc, Icariin, Catalpol, Rutin, Apigenin-7-glucoside, Wedelolactone, and Isoacteoside. As biological samples, they are characterized by small sampling volumes and relatively low drug concentrations. There are various endogenous substances in biological samples that may interfere with the determination; thus, the analytical methods established for pharmacokinetic studies need to meet conditions such as high selectivity and sensitivity. The Orbitrap Fusion MS technique was selected to investigate the different pharmacokinetic characteristics of 16 bioactive compounds (Figure 1 (**1**–**16**)) in JHGs in control and PF model rats.

## 2. Results and Discussion

### 2.1. Method Validation

The results of the specificity test are shown in Figure S1; the retention times of Paeonol, Nobiletin, Peimisine, Peiminine, Peimine, Cynaroside, Hesperidin, Ginsenoside Rb_1_, Ginsenoside Rb_2_, Ginsenoside Rc, Icariin, Catalpol, Tangeretin, Rutin, CBZ, Apigenin-7-*O*-d-glucoside, Wedelolactone, Baohuoside I, and Isoacteoside were 5.49 min, 7.81 min, and 2.99 min, respectively, 2.64 min, 2.58 min, 3.13 min, 3.41 min, 8.49 min, 8.76 min, 8.54 min, 6.17 min, 7.16 min, 8.44 min, 3.40 min, 5.33 min, 3.82 min, 5.47 min, 8.88 min, 3.23 min, respectively. With the comparison between the chromatogram of the quality control sample, the chromatogram of the sample, and the chromatogram of the blank plasma, it was observed that the separation was good, and the instrumental response was high. The results could illustrate that the plasma endogenous substances and other substances did not interfere with the determination of the components to be measured in rats. As shown in Table 1, the linearity of each component was good (r ≥ 0.9955). Table 1 listed the regression equation, linear range, and correlation coefficient. The intra-day and inter-day precision RSDs in Table 2 were less than 7.76%. The recovery was in the range of 80.37–102.72%, and the matrix effects of the components were in the range of 79.01–110.90% (Table 3), which did not affect the accurate quantification. Stability was in a good range (Table 4), which met the requirements for the analytical determination of biological samples. Overall, the established method was simple and rapid with high precision and good sensitivity, which could be used for the next research work.

### 2.2. Histopathological Examination of PF Model Rats

**Figure 2:**
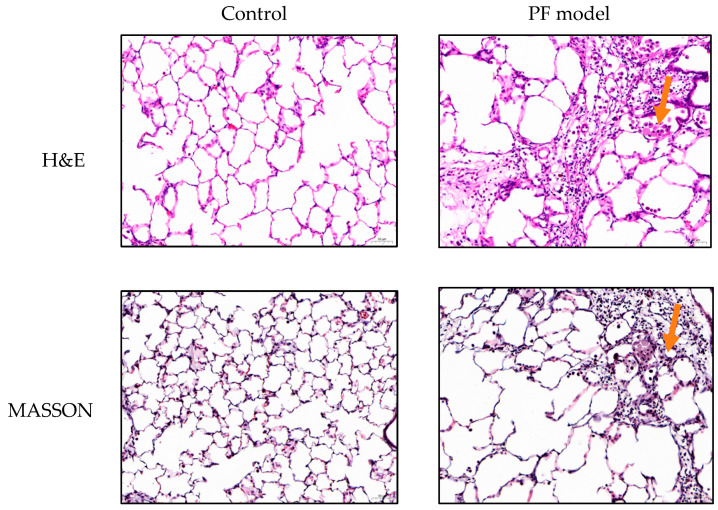
H&E and MASSON staining of control group and PF model group rats.

Under the light microscope (Figure 2), indicated by arrows, compared with the control group, the Hematoxylin-eosin (H&E) staining group observed alveolar wall thickening, inflammatory cell infiltration, alveolar structural disorder or destruction, diffuse hemorrhage, pale pink collagen fiber deposition, interstitial pneumonia, and other pulmonary solid changes. As for the MASSON group, the lung tissues showed collagen fiber deposition, continuous blue-stained areas, abnormal proliferation of fibroblasts, severe destruction of alveolar wall structure, and thickening of alveolar septa. The interstitial capillaries of lung tissue appeared to be bruised and dilated; the alveolar walls were thickened and even fractured. Inflammatory cells, mainly neutrophils, infiltrated the alveolar lumen. Thus, the construction of the PF model was well established.

### 2.3. Pharmacokinetics

#### 2.3.1. Pharmacokinetics Analysis of Control Group

**Figure 3:**
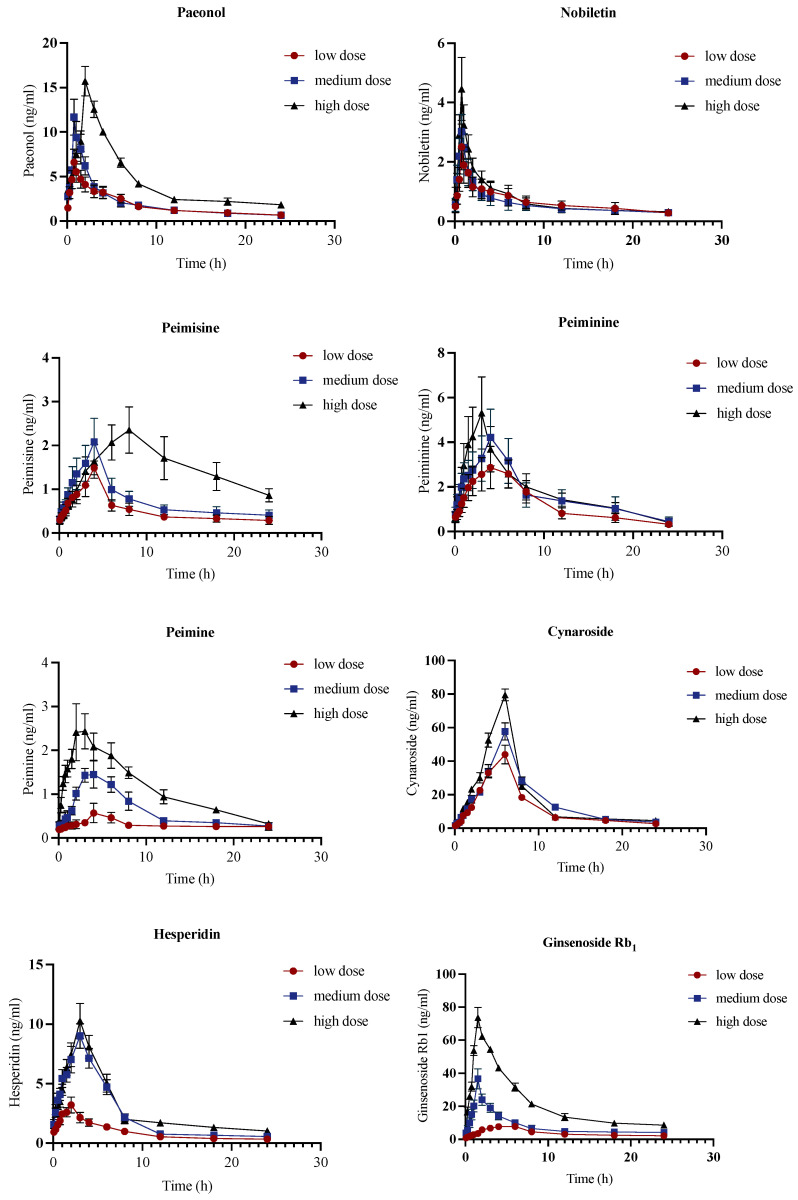

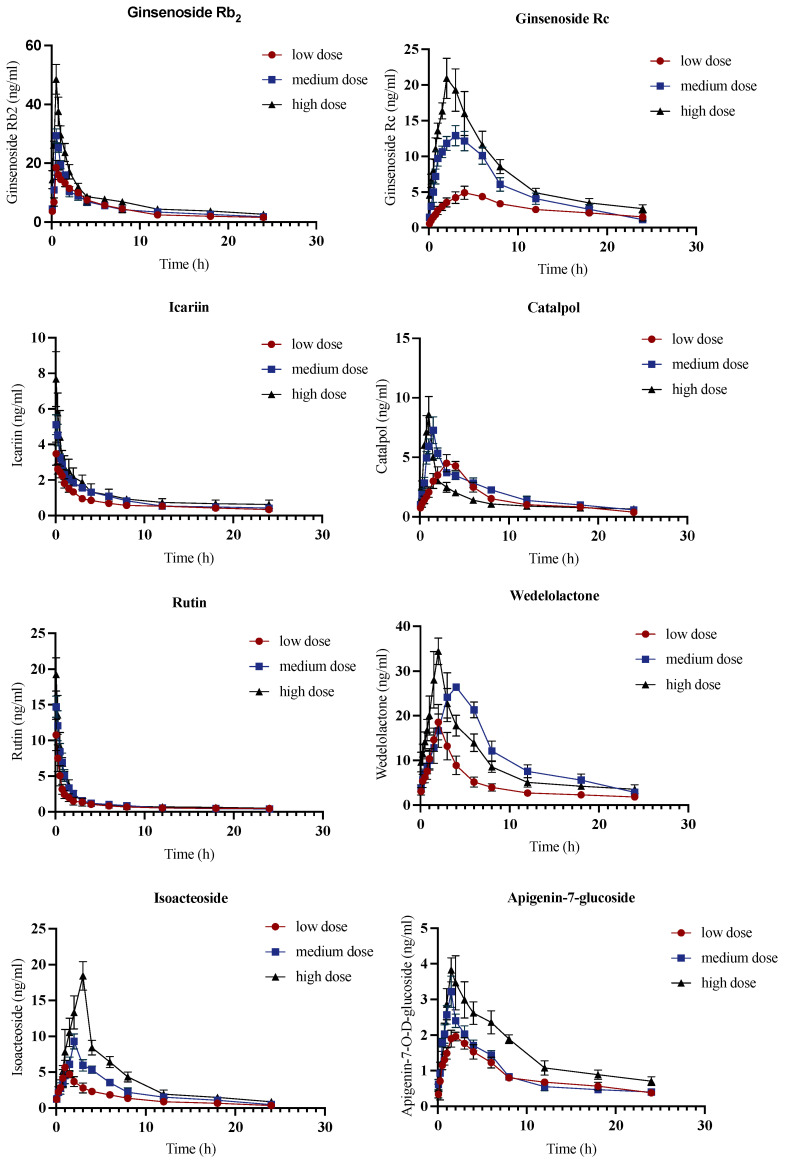
Concentration–time curves of control group of Paeonol, Nobiletin, Peimisine, Peiminine, Peimine, Cynaroside, Hesperidin, Ginsenoside Rb_1_, Ginsenoside Rb_2_, Ginsenoside Rc, Icariin, Catalpol, Rutin, Wedelolactone, Isoacteoside, and Apigenin-7-glucoside obtained after intragastric administration of JHG to control rats (*n* = 6). The vertical bars represent standard deviations.

The Orbitrap Fusion MS method was performed for a pharmacokinetic study of 16 components in control rats and PF model rats after intragastric of different doses (low, medium, high doses) of JHGs to determine the concentration according to the calibration curves. The concentration–time curves of the main bioactive compounds with three different concentrations in control groups are shown in Figure 3. The corresponding pharmacokinetic parameters of control group are shown in Table S1.

According to the pharmacokinetic parameters (Table S1), it was observed that Permisine, Peimine, Cynaroside, Ginsenoside Rb_1_, Ginsenoside Rb_2_, Ginsenoside Rc, Icariin and Isoacteoside in control rats followed the linear pharmacokinetic process. The C_max_ and AUCs increased with dosages. The rest of the compounds were non-linear, which could be attributed to the first-pass effect, absorption, distribution, metabolism, the excretion effect (ADME), and biotransformation [14,15,16]. Among them, there was a correlation between CL, AUCs (AUC_(0–t)_, AUC _(0–∞)_), t_½_, and dose for Cynaroside. For Rutin, there was no correlation between CL, AUCs, t_½_, and dose. T_max_ of all dosages of Paeonol, Nobiletin, Ginsenoside Rc, Icariin, Rutin and Apigenin-7-glucoside were achieved within 2 h after administration of JHGs, which indicated that these six compounds could be easily and rapidly absorbed in plasma, thus these six analytes could be the main effective compounds in JHG. However, the T_max_ of Cynaroside was the longest, no matter the dosages. Cynaroside had the highest AUCs among the analytes with a low administration dosage elucidating a marked bioavailability in the control group as well.

#### 2.3.2. Pharmacokinetics Analysis of PF Model Group

**Figure 4:**
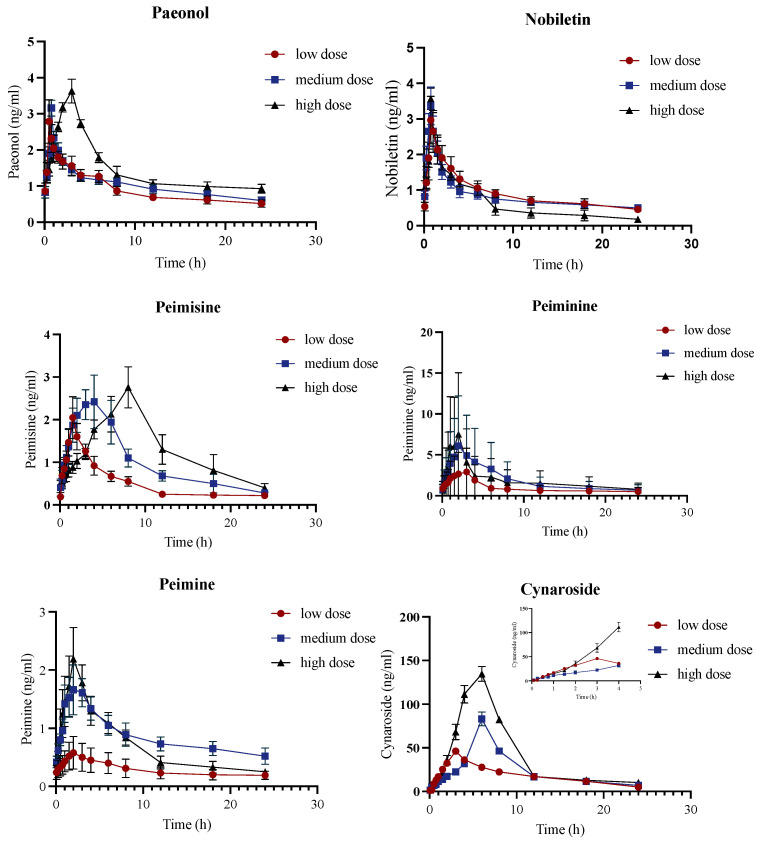

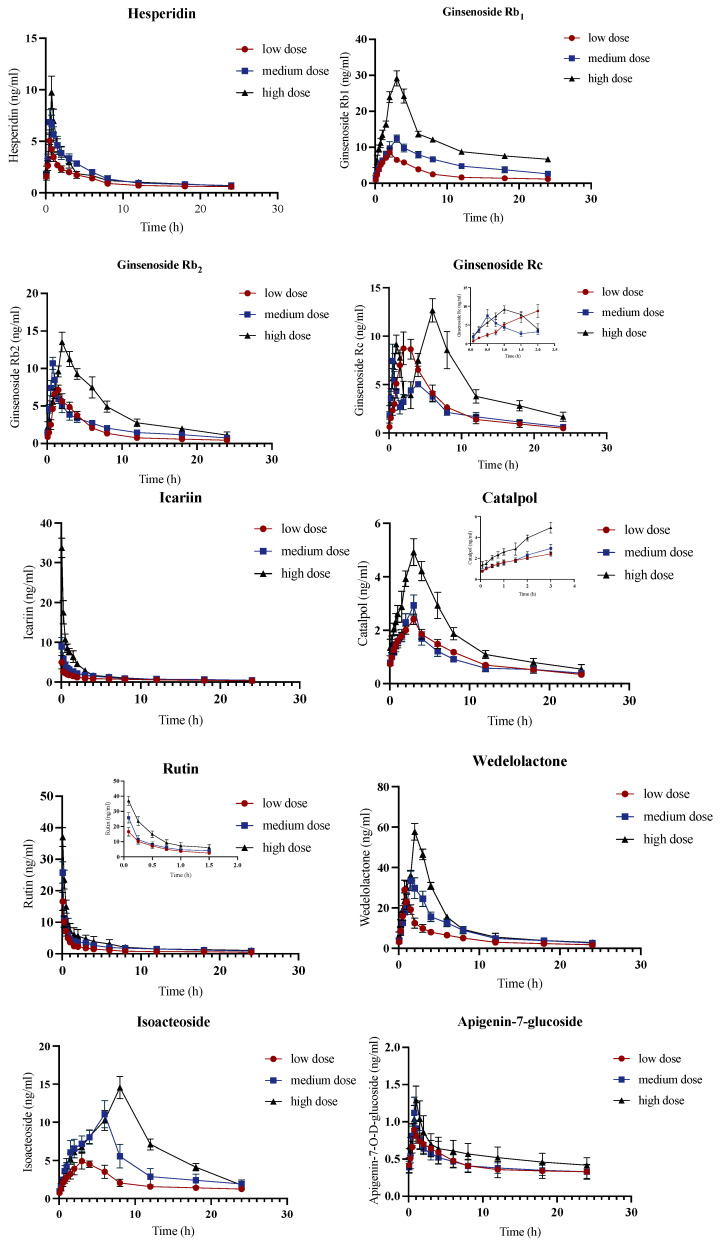
Concentration–time curves of PF model group of Paeonol, Nobiletin, Peimisine, Peiminine, Peimine, Cynaroside, Hesperidin, Ginsenoside Rb_1_, Ginsenoside Rb_2_, Ginsenoside Rc, Icariin, Catalpol, Rutin, Wedelolactone, Isoacteoside, and Apigenin-7-glucoside obtained after intragastric administration of JHGs to PF model rats (*n* = 6). The vertical bars represent standard deviations.

The concentration–time curves of the main bioactive compounds with three different concentrations in the PF model group are shown in Figure 4. The corresponding pharmacokinetic parameters of the PF model group are shown in Table S2. Many studies have shown that the pathological state might change the pharmacokinetic process of drugs. According to the pharmacokinetic parameters, it was observed that Permisine, Cynaroside, Ginsenoside Rb_1_, Ginsenoside Rb_2_, Rutin, Wedelolactone, and Isoacteoside in PF model rats followed the linear pharmacokinetic process. Among them, there was a correlation between CL, AUC, t_½_, and dose for Cynaroside. T_max_ of all dosages of Paeonol, Nobiletin, Ginsenoside Rb_2_, Icariin, Rutin, and Apigenin-7-glucoside was achieved within 2 h after administration of JHGs, with almost the same results as the control group, the pathological state did not have a large impact on their absorption rate. Cynaroside had the highest C_max_ and AUCs (AUC_(0–t)_, AUC _(0–∞)_) among the analytes with a low administration dosage, elucidating a marked bioavailability in the control group.

#### 2.3.3. Pharmacokinetics Comparison Analysis of Control and PF Model Groups

**Figure 5:**
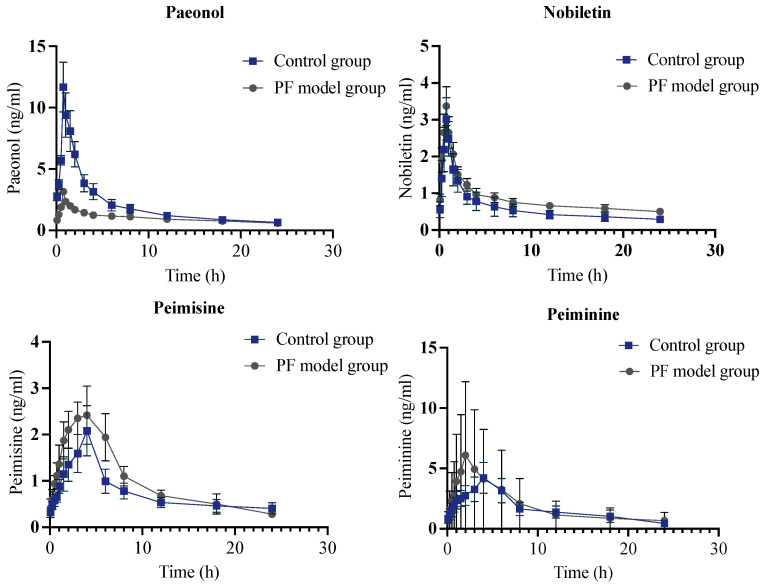

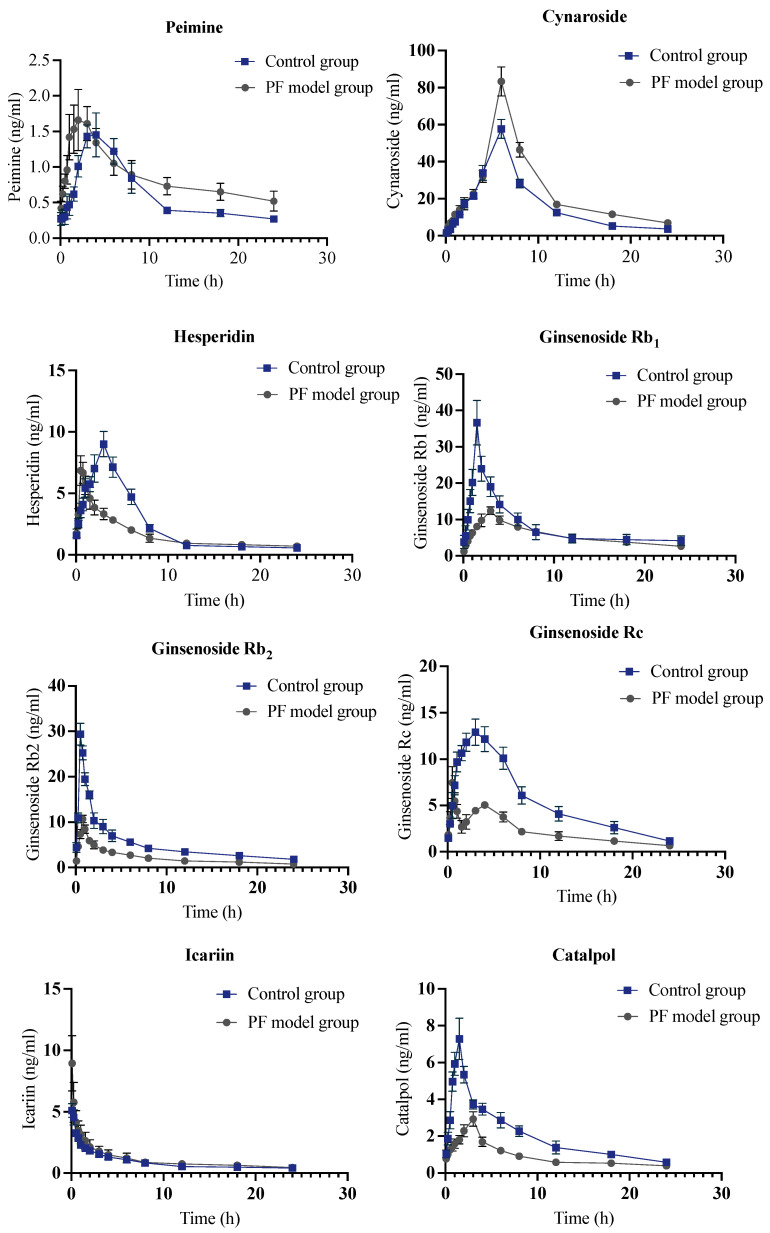

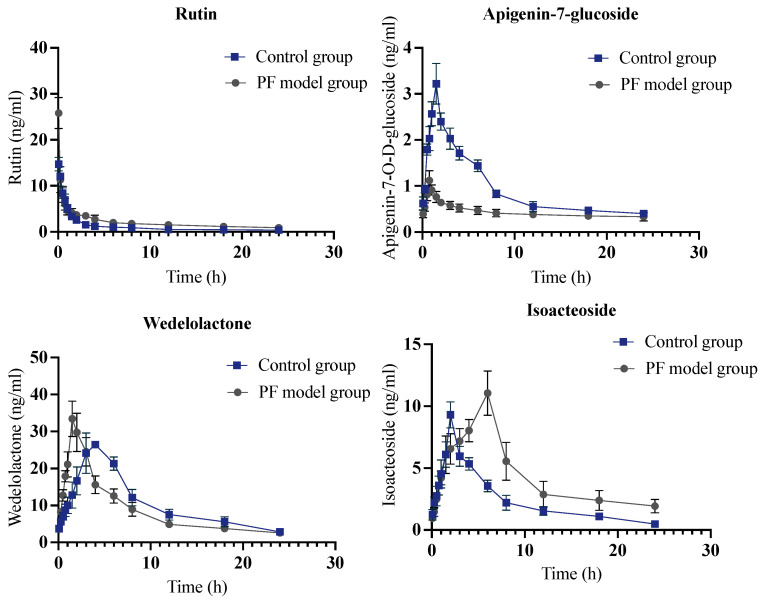
Medium dose of mean plasma concentration–time curves for control group and PF model group for Paeonol, Nobiletin, Peimisine, Peiminine, Peimine, Cynaroside, Hesperidin, Ginsenoside Rb_1_, Ginsenoside Rb_2_, Ginsenoside Rc, Icariin, Catalpol, Rutin, Apigenin-7 glucoside, Wedelolactone, and Isoacteoside obtained after intragastric administration of JHGs to control rats (*n* = 6). The vertical bars represent standard deviations.

A medium dose was selected to compare the differences between the PF model group and the control group, which was shown in Figure 5, to explore the pharmacokinetic tendency and research the variation of metabolic rate. Compared to the control group, the medium- and high-dose groups of Ginsenoside Rc exhibited a double peaks phenomenon in the PF model group, possibly due to enterohepatic circulation or other reasons.

Compared with the control group, the C_max_ of Paeonol, Ginsenoside Rb_1_, Ginsenoside Rb_2_, Catalpol, and Apigenin-7-glucoside were significantly decreased, while the C_max_ of Cynaroside, Icariin, Rutin, Peimine, Peimisine, Peiminine, and Wedelolactone were significantly increased, indicating that the pathological state of PF could have impacts on the absorption and metabolism of JHGs. Notably, the Peimine and the Peiminine were reported to inhibit lung inflammation and PF because of inhibiting inflammatory factors such as TNF-α, IL-6, IL-1β, IL-17 [17]. Peiminine inhibits the formation of lipid rafts that contribute to acute lung injury induced by lipopolysaccharide [18]. Peimisine, Peiminine, and Peimine were capable of being absorbed into the blood under physiological and pathological conditions [17,19]. It could thus elucidate that Peimisine, Peiminine, and Peimine could have a larger absorption in PF model group. As for Icariin, several studies have shown that Icariin has powerful anti-inflammatory and immunomodulatory properties, which could enhance immune defense and reduce lung infection risk [20]; thus, Icariin could have quite better bioavailability in the PF group compared to the control group. However, the intrinsic permeability of Icariin was dramatically poor [21,22]. This evidence could illustrate the slight increment of C_max_ of Icariin, which corresponded with the pharmacokinetic result. In particular, the complex interactions between the components in the herb prescription might influence the absorption of these ingredients in many aspects, such as the metabolism of gut microbiota and liver metabolism. The AUCs of Paeonol, Ginsenoside Rb_1_, Ginsenoside Rb_2_, and Apigenin-7-glucoside were significantly decreased (*p* < 0.05), indicating these compounds’ bioavailability declined. Accumulating evidence demonstrated that Peonol is rapidly absorbed from the gastrointestinal tract and rapidly distributed throughout the body, including the heart, brain, kidney, and liver, which has a short t_½_ and T_max_ after oral administration, which contributed to a poor bioavailability in vivo. The absorption of Paeonol occurs on a first-order basis without considering the concentration of the drug. Paeonol has a quick first-pass metabolism. However, in the PF model, the metabolism could be impacted, which could be a reason for the low bioavailability [23,24,25,26]. The T_max_ of Ginsenoside Rb_2_ was slightly increased in the PF model group. This increase varied among different doses, while the C_max_ was significantly reduced by about 1/3 comparatively. The AUCs decreased as well, with the control group elucidating that the pathological state of PF could lead to a slightly lower absorption rate and less absorption dosage. Though the Ginsenoside Rb_2_ respected a linear pharmacokinetic process, the oral administration of bioavailability was still low because of poor gastrointestinal absorption, resulting in low tissue-specific bioactivity. It was found that ginsenosides with large molecular masses, high hydrogen bond counts, and high molecular flexibility were less permeability to membranes, which probably elucidated the diminution of bioavailability of Ginsenoside Rb_2_ [27,28]. For Rutin, it has been shown to target various inflammatory, apoptotic, autophagic, and angiogenic signaling mediators, including nuclear factor-κB, tumor necrosis factor-α, interleukins, light chain 3/Beclin, B cell lymphoma 2 (Bcl-2), Bcl-2 associated X protein, caspases, and vascular endothelial growth factor [29]. The AUCs of Cynaroside and Rutin were significantly increased (*p* < 0.05), indicating a higher dosage of JHGs reached in system circulation; Rutin resulted in a benefit for the PF model rats. The T_max_ of Hesperidin, Peiminine, and Apigenin-7-glucoside decreased significantly, signaling that the disease state caused them to be absorbed dramatically faster. The results suggest that activating AMPK with Wedelolactone followed by reducing TGFf1/Raf-MAPK signaling pathways may have therapeutic potential for pulmonary fibrosis [30,31]. The T_max_ and C_max_ of Apigenin-7-glucoside in the model group were significantly reduced, indicating that the disease state accelerated the absorption of Apigenin-7-glucoside and reduced the absorption amount. The T_max_ of Isoacteoside in the model group increased about three times, which shows that the disease state affected the absorption and elimination of this component. The MRT_(0–t)_ of Peimisine, Peimine, and Wedelolactone decreased, possibly contributing to a delayed onset of drug action. Cynaroside is a flavonoid-like compound that was primarily hydrolyzed to luteolin, a flavonoid aglycone in the gastrointestinal tract absorbed into the systemic circulation [32,33]. The C_max_ and AUC_(0–t)_ of Cynaroside in the model group were approximately twice as high as those in the control group, illustrating that the disease state caused a significant increase in the uptake of Cynaroside.

Research of pharmacokinetic studies of Shen-Wu-Yi-Shen tablets (albiflorin, paeoniflorin, etc.) indicated the pharmacokinetic characteristics in normal and chronic renal failure exhibited different pharmacokinetic properties [34]. The results of comparative pharmacokinetics of Enmein, Epinodosin, and Isodocarpin indicated that three diterpenoids of Rabdosia serra Extract were significantly different between control and liver injury rats, which elucidated evident differences in the pharmacokinetic behaviors of compounds between the physiological and pathological states [16]. Moreover, the results of the pharmacokinetics of Xuanfei Baidu granules showed that pathological state promoted the absorption of several bioactive compounds, and pharmacokinetic behaviors changed in the ARDS rats model [35,36]. After taking a page from the research above, our results correspond with the pharmacokinetic study. Pathological states had an impact on JHG absorption.

## 3. Materials and Methods

### 3.1. Chemicals and Reagents

Paeonol Control (must-16071405, purity: 99.97%), Nobiletin Control (must-16070901, purity: 99.46%), Cynaroside Control (CHB151113, purity ≥ 98%), Hesperidin Control (must-16041806, purity: 99.70%), Peimisine Control (must-19072508, purity: 98.76%), and Isoacteoside Control (must-19103104, purity: 99.16%) were purchased from Chengdu Must Biotechnology Co., Ltd. (Chengdu, China) Peiminine Control (110751-201712, purity: 99.90%), Peimine Control (110750-201612, purity: 96.20%), Ginsenoside Rb_1_ Control (110704-201726, purity: 91.10%), Icariin Control (110737-201516, purity: 94.20%), and Rutin Control (100080-201408, Purity: 92.8%) were purchased from the China Academy of Food and Drug Administration. Ginsenoside Rb_2_ Control (131014, purity ≥ 98%), Ginsenoside Rc Control (131015, purity ≥ 98%), Catalpol Control (CHB181129, Purity: 98%), and Apigenin-7-glucoside Control (CHB161226, purity ≥ 98%) were purchased from Chengdu Chroma-Biotechnology Co., Ltd. (Chengdu, China) Wedelolactone (PJ0629RA13, purity ≥ 98%) and CBZ Control (H21M7L11256, purity ≥ 98%) were purchased from Shanghai Yuanye Bio-Technology Co. Ltd. (Shanghai, China) Bleomycin (BLM) hydrochloride (H20055883) was purchased from Pfizer Pharmaceutical Company Limited. Mass spectrometry methanol (TEDIA, Fairfield, OH, USA), mass spectrometry formic acid (Thermo Fisher Scientific, Waltham, MA, USA), and all other reagents were analytically pure, and the water was Milli-Q homemade ultrapure water. CBZ (Figure 1 (**17**)) was selected as the internal standard, which has the advantages of a stable nature and strong detection signal and can be completely separated from the measured substance. Hematoxylin-eosin (H&E) staining, and Masson’s Trichrome staining related reagent paraformaldehyde (P804536) were provided by Shanghai McLean Biochemical Technology Co., Ltd. (Shanghai, China). Paraffin wax (39601006) was provided by Leica Microsystems Trading Co. (Buffalo Grove, IL, USA). Anhydrous ethanol (500 mL) and xylene (500 mL) were provided by Tianjin Zhiyuan Chemical Reagent Co. (Tianjin, China) Hematoxylin staining solution (G1140), eosin staining solution (G1100), and neutral gum (G8590) were provided by Beijing Solepol Science and Technology Co., Ltd. (Beijing, China).

### 3.2. Instruments

UPLC-Orbitrap Fusion Mass Spectrometer, Xcalibur mass spectrometry workstation, SPD2010-230 SpeedVac centrifuge concentrator, 933 type ultra-low temperature refrigerator and Heraeus Multifuge X_1_R high-speed benchtop frozen centrifuge (Thermo Fisher Scientific, Waltham, MA, USA). MS105DU 1-in-100,000 analytical balance (Mettler, Zürich, Switzerland), Eppendorf pipette gun (Eppendorf, Shanghai, China), KH-250E Ultrasonic cleaner (Kunshan Wochuang Ultrasonic Instrument Co., Ltd., Kunshan, China), BE-3100 type super mixing elf (Haimen Qilinbeier Instrument Manufacturing Co., Ltd., Haimen, China), XH-C type vortex mixer (Jintan Guowang Experimental Instrument Factory, Jintan, China), KQ-500B type ultrasonic cleaner (Kunshan Ultrasonic Instrument Co., Ltd., Kunshan, China), Milli-QPOD ultrapure water preparer (Merck, Darmstadt, Germany).

### 3.3. Experimental Animals

Healthy male Sprague Dawley (SD) rats of SPF grade (240 ± 20 g) were purchased from Sipeifu (Beijing, China) Biotechnology Co., Ltd. (Beijing, China), Certificate of Conformity No.: SCYK (Beijing, China) 2019-0015, Animal Quality Certificate No.: 110324220102348616. They were housed in the Animal Center of Henan University of Traditional Chinese Medicine, which passed the ethical audit, ethics number: DWLL202208003, breeding Co. 24 °C, relative humidity 50 ± 2%. The animals fasted for 12 h before the experiment and drank water freely. All feeding and experimental studies on experimental animals were in accordance with the regulations on the Management of Experimental Animals in Henan Province.

### 3.4. Construction of the PF Model Rats

The PF model in rats was replicated by reference to the conclusion tracheal intubation method in the literature. After the rats were anesthetized, bleomycin (BLM) (5 mg/kg) was used as an induction agent and followed the tracheal intubation method. BLM solution was aspirated with a 1 mL syringe and injected into the indwelling syringe. A total of 0.3 mL of air was rapidly pushed into the syringe, the rats were immediately stroked upright so that the BLM solution was evenly distributed in both lungs. BLM was given on day 0 for modeling, then gavage was taken after day 28, with fasting without water for 12 h before sampling. The histopathological examination was performed to verify the construction of the PF model. The rats were anesthetized by the abdominal cavity, then the lung tissues were violently shocked by opening the chest, and the right lower lobe of the lung was taken and fixed in 4% formaldehyde solution, then dehydrated by sucrose gradient, paraffin-embedded, and sectioned. H&E and Masson staining were performed to observe the degree of alveolitis of lung tissue and the degree of pulmonary fibrosis, respectively. The inflammation and fibrosis of lung tissues was observed under the light microscope.

### 3.5. Preparation of Jinshui-Huanxian Granules

We add *Panax ginseng* C. A. Mey. and *Ophiopogon japonicus* (Thunb.) Ker Gawl., Rehmania glutinosa (Gaertn.) DC., Trichosanthes kirilowii Maxim., *Glycyrrhiza glabra* L. We added 12 times the amount of water and decocted twice, 1 h each time, filtered through, concentrated the filtrate, and set aside. Then, we added *Paeonia officinalis* L., Citrus × aurantium f. deliciosa (Ten.) M.Hiroe, *Ginkgo biloba* L., Fritillaria thunbergii Miq., *Epimedium brevicornu* Maxim, added 10 times the amount of 70% ethanol and refluxed twice (1 h each time). We filtered through, recovered the filtrate with no alcoholic smell, combined with the above-concentrated solution, continued to concentrate to a thick paste with a relative density of 1.18–1.22, dry at 60 °C, and crushed into fine powder. We added an appropriate amount of dextrin, mix well, using 80% ethanol as a wetting agent, made granules, and dried at 60 °C. Each 1g of Jinshui-Huanxian granules was equivalent to 1.89 g of raw drug, which contained 3161.16 μg of Paeonol, 201.68 μg of Nobiletin, 1.66 μg of Peimisine, 51.13 μg of Peiminine, 27.32 μg of Peimine, 12.02 μg of Cynaroside, 3077.18 μg of Hesperidin, 463.87 μg of Ginsenoside Rb_1_, 430.85 μg of Ginsenoside Rb_2_, 431.51 μg of Ginsenoside Rc, 608.93 μg of Icariin, 6.22 μg of Catalpol, 9.55 μg of Rutin, 3.52 μg of Apigenin-7-glucoside, 0.27 μg of Wedelolactone, and 19.68 μg of Isoacteoside. Finally, the Total Ion Current (TIC) chromatogram in Positive and Negative modes was performed; the chromatograms are shown in Figures S2 and S3.

### 3.6. Preparation of Control Solution

Measure 7.60 mg of Paeonol Control, 8.16 mg of Nobiletin Control, 8.00 mg of Peimisine Control, 7.72 mg of Peiminine Control, 8.04 mg of Peimine Control, 8.72 mg of Cynaroside Control, 7.72 mg of Hesperidin Control, 8.12 mg of Ginsenoside Rb_1_ Control, 7.76 mg of Ginsenoside Rb_2_ Control, 7.80 mg of Ginsenoside Rc Control, 8.68 mg of Icariin Control, 8.64 mg of Catalpol Control, 8.08 mg of Rutin Control, 7.52 mg of Apigenin-7-glucoside Control, 7.84 mg of Wedelolactone Control, 8.64 mg of Isoacteoside Control. Put them in a 10 mL flask, added methanol to dissolve them by sonication, diluted to 10 mL, and shake well. Add methanol and dilute to the scale of 10 mL, shake well, and use as the control stock solution. Weigh 7.80 mg of carbamazepine (CBZ) in a 10 mL volumetric flask, add methanol, diluted to the scale, shake well, measure 1 mL in a 10 mL volumetric flask, add methanol to dilute to the scale, and reserve as the internal standard solution.

### 3.7. Administration of Control Rats and PF Model Rats

Twenty-four healthy male SD rats or PF model rats, weighing 240 ± 20 g, fasted for 12 h before the experiment and drank water freely. Both kinds of rats were randomly divided into four groups. Blank group: equal dose of water by intragastric, low dose; administration group: rats were given the equivalent human dose of JHGs by intragastric, i.e., 10.8 g of raw drug/kg/d (5.70 g/kg/d); medium-dose administration group: 2 times the human equivalent dose of JHGs by intragastric, i.e., 21.6 g of raw drug/kg/d (11.41 g/kg/d); high-dose administration group: 4 times the human equivalent dose of JHG, i.e., 43.2 g of raw drug/kg/d (22.81 g/kg/d). Blood was collected from the tail vein before and 0.083, 0.25, 0.5, 0.75, 1, 1.5, 2, 3, 4, 6, 8, 12, 18, and 24 h after dosing, respectively. A total of 0.5 mL was placed in a heparin sodium centrifuge tube. Whole blood was left for 30 min and then centrifuged in a low-temperature freezing centrifuge (4 °C, 3000 r/min) for 15 min. The upper layer of plasma was collected and stored in a refrigerator at −80 °C.

### 3.8. Pretreatment of Biological Samples

We added 50 μL of internal standard solution and 800 μL of methanol, shook for 15 min, then centrifuged at 12,000 r/min for 10 min, took the supernatant, dried in a centrifuge concentrator, added 50 μL of the initial mobile phase to the residue, dissolved, centrifuged, then separated the upper layer of the solution used as the test solution. A total of 5 μL test solution was taken into the sample for analysis. For each compound with a certain dosage, the same procedure is repeated six times in parallel. The concentration in plasma was calculated by the internal standard method.

### 3.9. Chemical Profiling of Plasma Obtained after JHG Administration

#### 3.9.1. Chromatographic Condition

Accucore C_18_ (100 mm × 2.1 mm, 2.6 μm) column at 25 °C, the mobile phase was methanol (A)-0.1% formic acid aqueous solution (B). The gradient elution program was 0–4 min (65% B-40% B), 4–5 min (40% B-40% B), 5–6 min (40% B-28% B), and 6–7 min (28% B-19% B), 7–7.5 min (19% B-10% B), 7.5–11 min (10% B-0% B), the flow rate was 0.2 mL/min, and the injection volume was 5 μL.

#### 3.9.2. Mass Spectrometry Condition

The ion source was an electrospray ionization source (ESI). Nitrogen acted as a carrier gas. The sheath gas pressure was 35 Arb. The auxiliary gas pressure was 7 Arb. The spray voltage was 3.50 kv(+), 2.50 kv(-). The ion transport tube temperature was regulated at 300 °C. Gasification temperature was 275 °C. The resolution was 50,000. The scanning mode was set as SIM mode. The optimized parameters obtained are in Table 5.

### 3.10. Method Validation

The method validation satisfied linearity, specificity, sensitivity, precision, accuracy, recovery, matrix effect, and stability. Specificity was identified for the potential interferences. of compounds and internal standard by analyzing blank plasma samples. The calibration curves for quantitative analysis were calculated by plotting the peak area ratio (y) of each compound to IS against the corresponding nominal concentration (x), using weighted (1/x^2^) least-squares linear regression. The precision and accuracy were evaluated using 6 parallel quality control (QC) samples on three consecutive days. The intra- and inter-day accuracy and precision variations were represented by the relative error (RE) and relative standard deviation (RSD). The stability test of quality control (QC) samples in rat blank plasma was applied under 4 different concentrations in 3 different store environments, which were stored at 4 °C for 12 h, freezing at −80 °C for 30 days, and subjected to three freeze–thaw cycles from −20 °C to room temperature. Recovery was determined at four QC levels and calculated by comparing the analyte standard peak areas obtained from extracted samples with post-extracted samples spiked with the analytes. Matrix effects were calculated by matching spiking post-extracted blank plasma samples with corresponding standard clean solutions at three concentrations of QC samples.

### 3.11. Statistical Analyses

Under the established Orbitrap Fusion MS method, the pharmacokinetics of JHG in rats were studied to investigate the dynamic changes of the active components in rats. The pharmacokinetic parameters of each component in each dosing group were calculated by non-atrial model analysis using Kinetica 5.1 (Innaphase, Waltham, MA, USA) pharmacokinetic analysis software. The pharmacotemporal curves were plotted by the plotting software. SPSS 19.0 (IBM, Armonk, NY, USA) was used to analyze all the parameters. Independent sample tests were performed after natural logarithmic transformation for AUC_0–t_, C_max_. The nonparametric Mann–Whitney test was applied for T_max_, t_½_, and MRT_0–t_. *p* < 0.05 was considered statistically significant.

## 4. Conclusions

In summary, a reliable, rapid, stable Orbitrap Fusion MS method was established to determine the concentration of 16 main bioactive components of JHG in PF model and control rats. Different pharmacokinetic properties of the 16 bioactive compounds between control and PF model rats were observed. Pathologies state induced by BLM performed a remarkable effect on pharmacokinetics in vivo. Above all, this study provides an indispensable reference for exploring the mechanism of absorption of Jinshui-Huanxian granules and provides a guiding clinical medicine.

## Figures and Tables

**Table 1:** Regression equation, linear range, and correlation coefficient of 16 main bioactive compounds.

Compounds	Calibration Curves	Liner Range (ng/mL)	r
Paeonol	y = 0.00460 x − 0.00816	0.74–380.00	0.9995
Nobiletin	y = 0.10179 x + 1.66932	0.40–204.00	0.9969
Peimisine	y = 0.09487 x + 0.64909	0.39–200.00	0.9974
Peiminine	y = 0.09893 x + 0.85198	0.75–386.00	0.9971
Peimine	y = 0.08954 x + 1.13652	0.39–201.00	0.9963
Cynaroside	y = 0.01271 x + 0.12944	1.70–872.00	0.9970
Hesperidin	y = 0.01078 x − 0.00926	0.75–386.00	0.9989
Ginsenoside Rb_1_	y = 0.00328 x + 0.02746	0.79–406.00	0.9955
Ginsenoside Rb_2_	y = 0.00286 x + 0.08165	0.76–388.00	0.9968
Ginsenoside Rc	y = 0.00442 x + 0.15620	0.76–390.00	0.9970
Icariin	y = 0.01509 x + 0.11806	0.42–217.00	0.9979
Catalpol	y = 0.00271 x − 0.00685	0.42–216.00	0.9994
Rutin	y = 0.01117 x + 0.04823	0.79–404.00	0.9974
Apigenin-7-glucoside	y = 0.01300 x + 0.18093	0.37–188.00	0.9964
Wedelolactone	y = 0.00743 x + 0.05249	0.77–392.00	0.9955
Isoacteoside	y = 0.00152 x − 0.01238	0.42–216.00	0.9972

**Table 2:** Precision and accuracy of 16 main bioactive compounds in rat plasma ($\bar{X}$ ± S, *n* = 6).

Compounds	Concentration (ng/mL)	Intra-Day			Inter-Day		
		Mean ± SD (ng/mL)	Accuracy (RE, %)	Precision (RSD, %)	Mean ± SD (ng/mL)	Accuracy (RE, %)	Precision (RSD, %)
Paeonol	2.97	2.93 ± 0.06	−1.35	2.05	2.93 ± 0.09	−1.35	3.07
	11.88	11.87 ± 0.13	−0.08	1.10	12.03 ± 0.15	1.26	1.25
	95.00	95.75 ± 3.56	0.79	3.72	94.91 ± 4.04	−0.09	4.26
Nobiletin	1.59	1.56 ± 0.06	−1.89	3.85	1.58 ± 0.06	−0.63	3.80
	6.38	6.4 ± 0.15	0.31	2.34	6.42 ± 0.25	0.63	3.89
	51.00	51.32 ± 2.2	0.63	4.29	49.59 ± 3.85	−2.76	7.76
Peimisine	1.56	1.54 ± 0.04	−1.28	2.60	1.6 ± 0.05	2.56	3.13
	6.25	6.31 ± 0.16	0.96	2.54	6.31 ± 0.05	0.96	0.79
	50.00	50.53 ± 2.05	1.06	4.06	50.31 ± 3.43	0.62	6.82
Peiminine	3.02	3.02 ± 0.1	0.00	3.31	3.01 ± 0.09	−0.33	2.99
	12.06	12.19 ± 0.05	1.08	0.41	12.22 ± 0.32	1.33	2.62
	96.50	96.74 ± 2.68	0.25	2.77	94.45 ± 2.16	−2.12	2.29
Peimine	1.57	1.56 ± 0.02	−0.64	1.28	1.56 ± 0.04	−0.64	2.56
	6.28	6.47 ± 0.09	3.03	1.39	6.37 ± 0.34	1.43	5.34
	50.25	51.89 ± 1.82	3.26	3.51	51.28 ± 3.6	2.05	7.02
Cynaroside	6.81	6.92 ± 0.22	1.62	3.18	6.9 ± 0.2	1.32	2.90
	27.25	27.62 ± 1.04	1.36	3.77	27.74 ± 1.21	1.80	4.36
	218.00	215.85 ± 4.75	−0.99	2.20	216.04 ± 7.9	−0.90	3.66
Hesperidin	3.02	3.13 ± 0.14	3.64	4.47	3 ± 0.13	−0.66	4.33
	12.06	11.98 ± 0.12	−0.66	1.00	12.23 ± 0.22	1.41	1.80
	96.50	96.04 ± 1.12	−0.48	1.17	92.88 ± 0.93	−3.75	1.00
Ginsenoside Rb_1_	3.17	3.13 ± 0.12	−1.26	3.83	3.21 ± 0.06	1.26	1.87
	12.69	12.53 ± 0.17	−1.26	1.36	12.53 ± 0.75	−1.26	5.99
	101.50	100.11 ± 1.74	−1.37	1.74	101.54 ± 2.93	0.04	2.89
Ginsenoside Rb_2_	3.03	2.99 ± 0.08	−1.32	2.68	2.97 ± 0.16	−1.98	5.39
	12.13	11.87 ± 0.13	−2.14	1.10	12.26 ± 0.4	1.07	3.26
	97.00	97.46 ± 1.71	0.47	1.75	95.55 ± 2.16	−1.49	2.26
Ginsenoside Rc	3.05	3.08 ± 0.11	0.98	3.57	3.02 ± 0.08	−0.98	2.65
	12.19	12.04 ± 0.25	−1.23	2.08	12.31 ± 0.21	0.98	1.71
	97.50	98.22 ± 1.49	0.74	1.52	96.84 ± 2.78	−0.68	2.87
Icariin	1.70	1.66 ± 0.11	−2.35	6.63	1.67 ± 0.04	−1.76	2.40
	6.78	6.58 ± 0.15	−2.95	2.28	6.89 ± 0.22	1.62	3.19
	54.25	51.94 ± 1.22	−4.26	2.35	54.38 ± 1.43	0.24	2.63
Catalpol	1.69	1.7 ± 0.13	0.59	7.65	1.69 ± 0.05	0.00	2.96
	6.75	6.59 ± 0.24	−2.37	3.64	6.85 ± 0.2	1.48	2.92
	54.00	54.51 ± 1.92	0.94	3.52	53.73 ± 2.28	−0.50	4.24
Rutin	3.16	3.14 ± 0.1	−0.63	3.18	3.21 ± 0.09	1.58	2.80
	12.63	12.6 ± 0.18	−0.24	1.43	12.31 ± 0.51	−2.53	4.14
	101.00	101.76 ± 1.68	0.75	1.65	100.42 ± 3.67	−0.57	3.65
Apigenin-7-glucoside	1.47	1.52 ± 0.05	3.40	3.29	1.51 ± 0.03	2.72	1.99
	5.88	5.94 ± 0.2	1.02	3.37	5.95 ± 0.16	1.19	2.69
	47.00	46.38 ± 1.8	−1.32	3.88	47.19 ± 1.49	0.40	3.16
Wedelolactone	3.06	3.11 ± 0.08	1.63	2.57	2.99 ± 0.08	−2.29	2.68
	12.25	11.93 ± 0.17	−2.61	1.42	12.5 ± 0.14	2.04	1.12
	98.00	98.01 ± 1.34	0.01	1.37	100.16 ± 4.37	2.20	4.36
Isoacteoside	1.69	1.74 ± 0.03	2.96	1.72	1.65 ± 0.05	−2.37	3.03
	6.75	6.63 ± 0.27	−1.78	4.07	6.65 ± 0.14	−1.48	2.11
	54.00	54.03 ± 1.34	0.06	2.48	52.27 ± 1.76	−3.20	3.37

RE: relative error (%) = [(measured concentration − nominal concentration)/nominal concentration] × 100; RSD: relative standard deviation = (standard deviation/mean deviation) × 100.

**Table 3:** Recovery and matrix effects of the 16 main bioactive compounds in rat plasma ($\bar{X}$ ± S, *n* = 6).

Compounds	Concentration	Matrix Effect		Recovery	
	(ng/mL)	Mean ± SD (%)	RSD (%)	Mean ± SD (%)	RSD (%)
Paeonol	2.97	93.14 ± 5.09	5.46	85.27 ± 2.28	2.67
	11.88	98.88 ± 6.79	6.87	84.55 ± 2.86	3.38
	95.00	98.22 ± 3.67	3.74	93.2 ± 1.81	1.94
Nobiletin	1.59	96.56 ± 4.54	4.70	94.68 ± 2.38	2.51
	6.38	106.64 ± 2.58	2.42	97.51 ± 2.61	2.68
	51.00	103.78 ± 1.31	1.26	86.65 ± 6.09	7.03
Peimisine	1.56	93.63 ± 5.26	5.62	99.11 ± 2.45	2.47
	6.25	84.94 ± 6.23	7.33	91.58 ± 2.69	2.94
	50.00	93.78 ± 2.18	2.32	85.18 ± 4.54	5.33
Peiminine	3.02	100.22 ± 6.53	6.52	96.97 ± 0.74	0.76
	12.06	100.18 ± 3.75	3.74	96.55 ± 6.56	6.79
	96.50	99.6 ± 0.12	0.12	85.17 ± 3.5	4.11
Peimine	1.57	96.05 ± 5.72	5.96	87.64 ± 0.99	1.13
	6.28	92.11 ± 8.13	8.83	97.01 ± 4.23	4.36
	50.25	97.2 ± 0.19	0.20	88.13 ± 2.61	2.96
Cynaroside	6.81	103.89 ± 4.92	4.74	86.15 ± 4.19	4.86
	27.25	91.33 ± 1.38	1.51	86.72 ± 5.97	6.88
	218.00	99.35 ± 3.3	3.32	87.85 ± 2.27	2.58
Hesperidin	3.02	100.33 ± 1.89	1.88	95.3 ± 3.9	4.09
	12.06	84.75 ± 5.16	6.09	97.75 ± 2.87	2.94
	96.50	96.09 ± 1.72	1.79	93.26 ± 2.88	3.09
Ginsenoside Rb_1_	3.17	92.03 ± 2.51	2.73	94.95 ± 3.85	4.05
	12.69	92.27 ± 3.22	3.49	96.03 ± 5.26	5.48
	101.50	93.37 ± 5.07	5.43	86.15 ± 4.6	5.34
Ginsenoside Rb_2_	3.03	91.42 ± 6.33	6.92	88.04 ± 3.75	4.26
	12.13	94.61 ± 2.06	2.18	99.47 ± 2.21	2.22
	97.00	88.82 ± 7.48	8.42	82.7 ± 1.51	1.83
Ginsenoside Rc	3.05	91.44 ± 3.68	4.02	84.32 ± 3.29	3.90
	12.19	92.83 ± 1.11	1.20	97.59 ± 3.83	3.92
	97.50	95.49 ± 1.05	1.10	95.32 ± 2.86	3.00
Icariin	1.70	95.25 ± 2.17	2.28	92.05 ± 1.86	2.02
	6.78	85.18 ± 1.77	2.08	94.23 ± 2.5	2.65
	54.25	97.27 ± 0.54	0.56	98.33 ± 4.26	4.33
Catalpol	1.69	96.36 ± 4.96	5.15	100.08 ± 0.98	0.98
	6.75	101.64 ± 2.94	2.89	101.27 ± 0.77	0.76
	54.00	88.34 ± 5.33	6.03	100.31 ± 2.13	2.12
Rutin	3.16	99.04 ± 4.12	4.16	95.24 ± 1.98	2.08
	12.63	87.09 ± 1.66	1.91	99.95 ± 1.8	1.80
	101.00	92.62 ± 2.17	2.34	96.37 ± 2.39	2.48
Apigenin-7-glucoside	1.47	83.65 ± 3.02	3.61	82.96 ± 3.27	3.94
	5.88	104.84 ± 3.38	3.22	94.31 ± 6.08	6.45
	47.00	92.68 ± 2.76	2.98	99.82 ± 1.93	1.93
Wedelolactone	3.06	101.11 ± 3.14	3.11	97.09 ± 2.17	2.24
	12.25	90.24 ± 6.81	7.55	84.91 ± 3.13	3.69
	98.00	88.54 ± 1.53	1.73	94.01 ± 4.11	4.37
Isoacteoside	1.69	96.74 ± 1.66	1.72	85.51 ± 1.65	1.93
	6.75	103.15 ± 3.67	3.56	95.94 ± 3.66	3.81
	54.00	91.85 ± 5.1	5.55	97.87 ± 4.56	4.66

**Table 4:** The stability test of 16 main bioactive compounds in control rat plasma ($\bar{X}$ ± S, *n* = 6).

Compounds	Concentration (ng/mL)	4 °C for 12 h		−80 °C for 30 Days		Cycles −20 °C-RT	
		Mean ± SD	RSD	Mean ± SD	RSD	Mean ± SD	RSD
		(ng/mL)	(%)	(ng/mL)	(%)	(ng/mL)	(%)
Paeonol	2.97	2.99 ± 0.07	0.67	2.94 ± 0.08	−1.01	2.88 ± 0.09	−3.03
	11.88	11.6 ± 0.16	−2.36	11.64 ± 0.6	−2.02	11.43 ± 0.53	−3.79
	95.00	95.31 ± 1.69	0.33	95.58 ± 1.35	0.61	94.59 ± 0.45	−0.43
Nobiletin	1.59	1.56 ± 0.05	−1.89	1.54 ± 0.02	−3.14	1.6 ± 0.06	0.63
	6.38	6.34 ± 0.08	−0.63	6.26 ± 0.04	−1.88	6.28 ± 0.1	−1.57
	51.00	50.51 ± 1.82	−0.96	49.68 ± 0.46	−2.59	51.92 ± 0.32	1.80
Peimisine	1.56	1.57 ± 0.1	0.64	1.55 ± 0.12	−0.64	1.6 ± 0.1	2.56
	6.25	6.24 ± 0.05	−0.16	6.31 ± 0.07	0.96	6.22 ± 0.01	−0.48
	50.00	49.8 ± 1.37	−0.40	50.32 ± 1.57	0.64	50.66 ± 1.84	1.32
Peiminine	3.02	3.06 ± 0.03	1.32	2.94 ± 0.02	−2.65	3 ± 0.05	−0.66
	12.06	12.56 ± 0.23	4.15	11.61 ± 0.58	−3.73	11.73 ± 0.69	−2.74
	96.50	99.02 ± 0.8	2.61	98.96 ± 1.07	2.55	96.34 ± 2.48	−0.17
Peimine	1.57	1.62 ± 0.04	3.18	1.58 ± 0.03	0.64	1.55 ± 0.04	−1.27
	6.28	6.3 ± 0.08	0.32	6.3 ± 0.09	0.32	6.22 ± 0.01	−0.96
	50.25	50.96 ± 0.85	1.41	50.47 ± 0.94	0.44	51.18 ± 0.38	1.85
Cynaroside	6.81	6.78 ± 0.13	−0.44	6.73 ± 0.1	−1.17	6.69 ± 0.12	−1.76
	27.25	26.93 ± 0.7	−1.17	27.72 ± 0.59	1.72	27.03 ± 0.97	−0.81
	218.00	210.42 ± 0.41	−3.48	220.91 ± 5.82	1.33	217.75 ± 4.43	−0.11
Hesperidin	3.02	2.99 ± 0.07	−0.99	3.05 ± 0.03	0.99	3 ± 0.09	−0.66
	12.06	11.89 ± 0.37	−1.41	12.41 ± 0.77	2.90	11.88 ± 0.42	−1.49
	96.50	97.08 ± 1.53	0.60	95.96 ± 0.55	−0.56	98.23 ± 1.45	1.79
Ginsenoside Rb_1_	3.17	3.3 ± 0.06	4.10	3.26 ± 0.08	2.84	3.26 ± 0.07	2.84
	12.69	12.82 ± 1.04	1.02	12.68 ± 0.56	−0.08	12.82 ± 0.25	1.02
	101.50	97.93 ± 3.39	−3.52	99.37 ± 6.7	−2.10	98.16 ± 4.34	−3.29
Ginsenoside Rb_2_	3.03	3.01 ± 0.03	−0.66	2.99 ± 0.08	−1.32	3.01 ± 0.06	−0.66
	12.13	11.74 ± 0.53	−3.22	12.4 ± 0.31	2.23	12.05 ± 0.11	−0.66
	97.00	97.48 ± 1.87	0.49	97.81 ± 3.29	0.84	97.34 ± 2.3	0.35
Ginsenoside Rc	3.05	3 ± 0.08	−1.64	2.99 ± 0.09	−1.97	2.99 ± 0.08	−1.97
	12.19	12.1 ± 0.42	−0.74	11.67 ± 0.6	−4.27	11.93 ± 0.1	−2.13
	97.50	96.31 ± 1.54	−1.22	95.94 ± 1.26	−1.60	96.83 ± 2.34	−0.69
Icariin	1.70	1.67 ± 0.04	−1.76	1.69 ± 0.09	−0.59	1.73 ± 0.04	1.76
	6.78	6.74 ± 0.13	−0.59	6.76 ± 0.06	−0.29	6.74 ± 0.1	−0.59
	54.25	53.36 ± 0.39	−1.64	53.65 ± 0.85	−1.11	53.73 ± 0.6	−0.96
Catalpol	1.69	1.68 ± 0.08	−0.59	1.73 ± 0.08	2.37	1.64 ± 0.03	−2.96
	6.75	6.76 ± 0.06	0.15	6.68 ± 0.12	−1.04	6.82 ± 0.09	1.04
	54.00	53.79 ± 0.54	−0.39	53.82 ± 0.29	−0.33	53.63 ± 1.11	−0.69
Rutin	3.16	3.2 ± 0.08	1.27	3.22 ± 0.08	1.90	3.06 ± 0.03	−3.16
	12.63	12.89 ± 0.5	2.06	12.46 ± 0.12	−1.35	12.74 ± 0.18	0.87
	101.00	99.03 ± 2.76	−1.95	101.08 ± 5.18	0.08	98.97 ± 5.4	−2.01
Apigenin-7-glucoside	1.47	1.52 ± 0.04	3.40	1.52 ± 0.07	3.40	1.52 ± 0.08	3.40
	5.88	5.85 ± 0.11	−0.51	5.85 ± 0.16	−0.51	5.8 ± 0.06	−1.36
	47.00	46.72 ± 0.41	−0.60	47.37 ± 0.56	0.79	46.9 ± 0.55	−0.21
Wedelolactone	3.06	3.06 ± 0.13	0.00	3.04 ± 0.03	−0.65	3.05 ± 0.13	−0.33
	12.25	11.68 ± 0.45	−4.65	12.18 ± 0.69	−0.57	11.97 ± 0.69	−2.29
	98.00	98.18 ± 0.77	0.18	97.17 ± 0.77	−0.85	97.33 ± 2.51	−0.68
Isoacteoside	1.69	1.72 ± 0.05	1.78	1.71 ± 0.02	1.18	1.69 ± 0.06	0.00
	6.75	6.78 ± 0.01	0.44	6.76 ± 0.02	0.15	6.76 ± 0.05	0.15
	54.00	54.38 ± 0.62	0.70	53.27 ± 0.37	−1.35	53.32 ± 1.2	−1.26

RT: room temperature (°C).

**Table 5:** The mass spectrometry parameters of 16 bioactive compounds and IS.

Compounds	RT (min)	*m*/*z*	Ionization Mode
Paeonol	5.49	167.0702	[M + H]+
Nobiletin	7.81	403.1387	[M + H]+
Peimisine	2.99	428.3159	[M + H]+
Peiminine	2.64	430.3315	[M + H]+
Peimine	2.58	432.3472	[M + H]+
Cynaroside	3.13	449.1078	[M + H]+
Hesperidin	3.41	609.1824	[M - H]-
Ginsenoside Rb_1_	8.49	1109.6103	[M + H]+
Ginsenoside Rb_2_	8.76	1079.5996	[M + H]+
Ginsenoside Rc	8.54	1079.5996	[M + H]+
Icariin	6.17	677.2439	[M + H]+
Catalpol	7.16	363.1285	[M + H]+
Rutin	3.40	609.1461	[M - H]-
Apigenin-7-glucoside	3.82	431.0983	[M - H]-
Wedelolactone	5.47	313.0353	[M - H]-
Isoacteoside	3.23	623.1981	[M - H]-
Carbamazepine (IS)	5.33	237.1022	[M + H]+

## Supplementary Materials

The following supporting information can be downloaded at: https://www.mdpi.com/article/10.3390/molecules28186492/s1, Figure S1: Orbitrap Fusion MS of the 16 bioactive compounds for the specificity test; Figure S2: Total Ion Current (TIC) (Positive) of Jinshui-Huanxian granules (JHG); Figure S3: Total Ion Current (TIC) (negative) of Jinshui-Huanxian granules (JHG); Table S1: Main pharmacokinetic parameters of the control groups after intragastric administration of JHF; Table S2: Main pharmacokinetic parameters of the PF model group after intragastric administration of JHF.

## Sample Availability

Samples of the compounds are available from the authors.

## Abbreviations

JHG: Jinshui-Huanxian granules, PF: pulmonary fibrosis, CM: Chinese medicine, IPF: idiopathic pulmonary fibrosis, WHO: World Health Organization, BLM: bleomycin hydrochloride, IS: internal standard, RT: room temperature, ESI: electrospray ionization, MS: mass spectrometry, SD: Sprague Dawley, TIC: total ion current, QC: quality control, C_max_: peak drug concentration, AUC: area under the plasma concentration-time, MRT: mean retention time, CL: clearance, t_½_: elimination half-life, T_max_: maximum plasma concentration, ADME: absorption, distribution, metabolism and excretion effect, RE: relative error, RSD: relative standard deviation, H&E: Hematoxylin-eosin.
