# Supplementary material for: A Pharmacokinetic Study of Sixteen Major Bioactive Components of Jinshui-Huanxian Granules in Pulmonary Fibrosis Model and Control Rats Using Orbitrap Fusion Mass Spectrometry
Source: Molecules. 2023 Sep 7;28(18):6492. doi: 10.3390/molecules28186492 (PMC10534582; doi:10.3390/molecules28186492)
Supplement: Supplementary file 1 [file molecules-28-06492-s001.zip › molecules-2570591-supplementary.pdf]

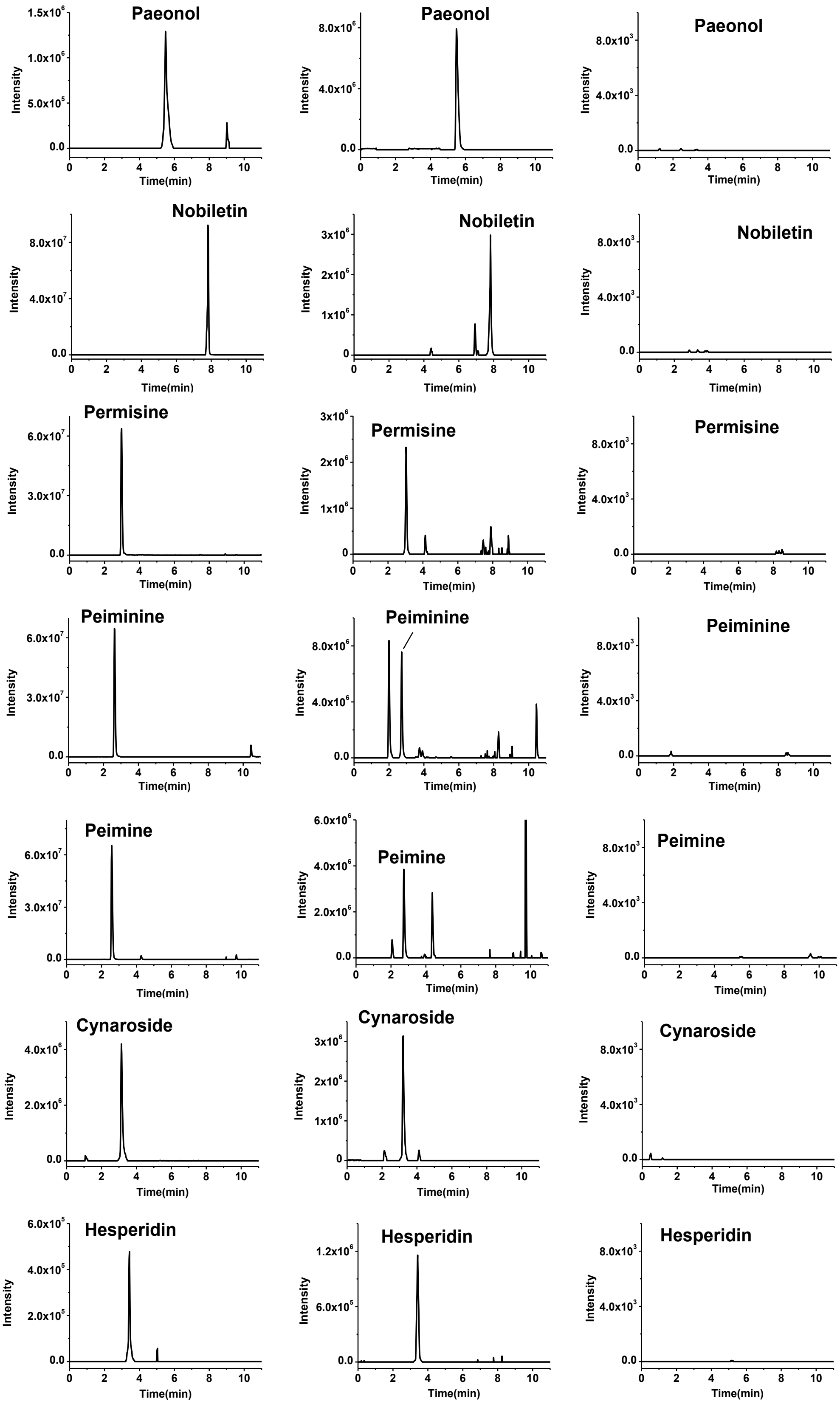
Paeonol
Paeonol
Paeonol
Nobiletin
Nobiletin
Nobiletin
Permisine
Permisine
Permisine
Peiminine
Peiminine
Peiminine
Peimine
Peimine
Peimine
Cynaroside
Cynaroside
Cynaroside
Hesperidin
Hesperidin
Hesperidin
Intensity
Time(min)

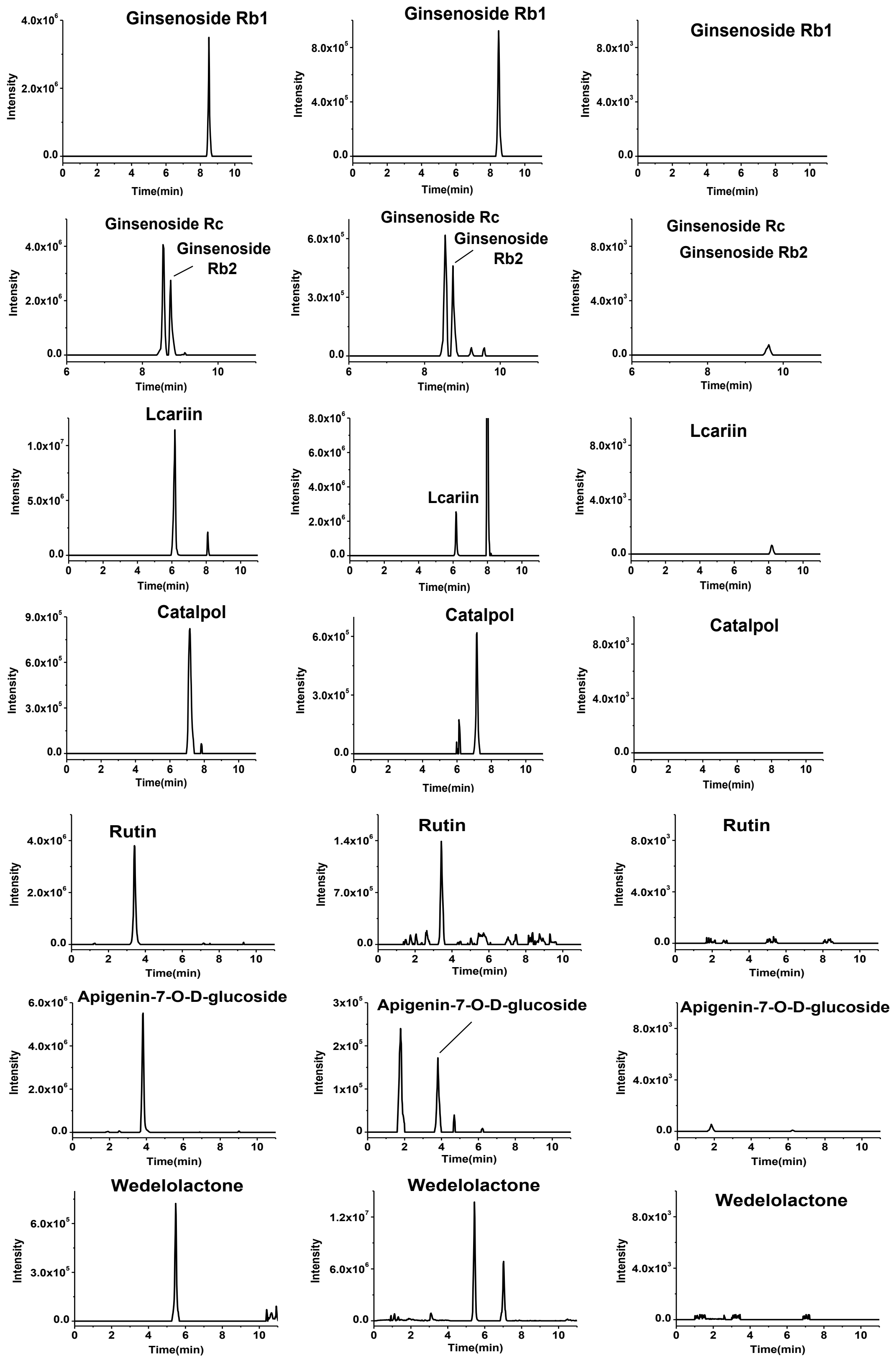
Ginsenoside Rb1
Ginsenoside Rc
Ginsenoside Rb2
Lcariin
Catalpol
Rutin
Apigenin-7-O-D-glucoside
Wedelolactone
Intensity
Time(min)

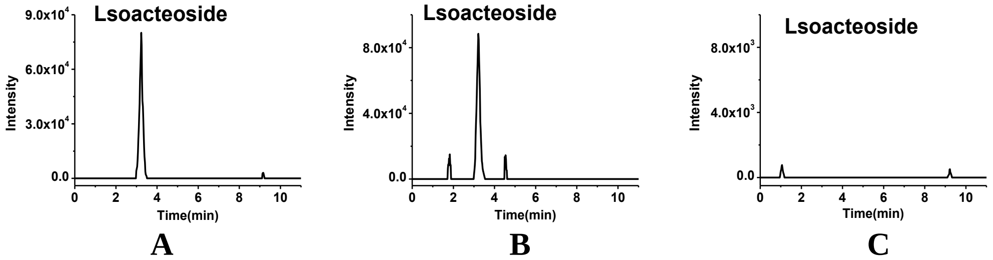

Fig. S1. Orbitrap Fusion MS of the 16 bioactive compounds for the specificity test
(A: chromatogram of quality control sample B: chromatogram of sample C: chromatogram of blank plasma)

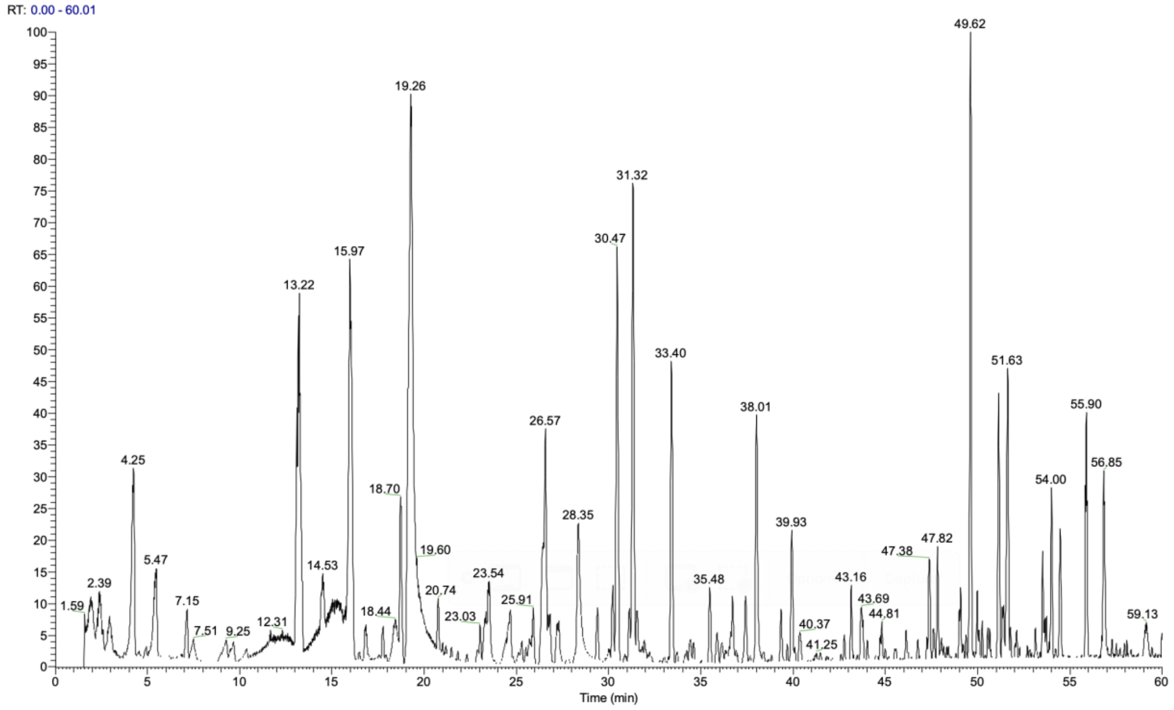

Fig. S2. Total Ion Current (TIC) (Positive) of Jinshui-Huanxian granules (JHG)

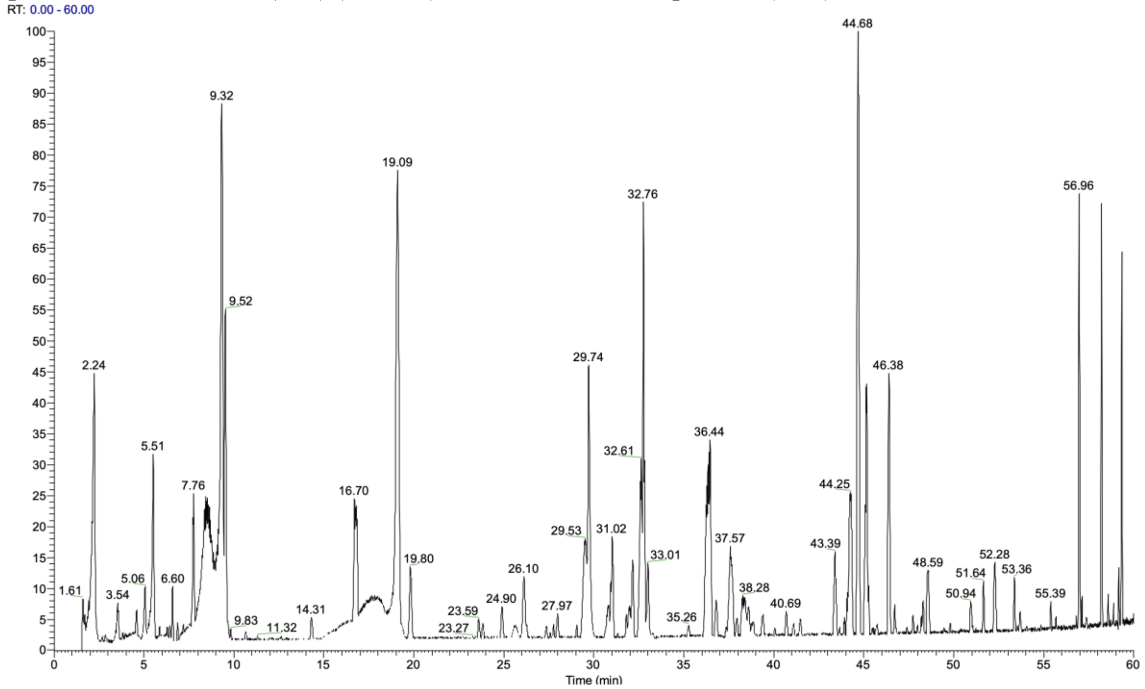

Fig. S3. Total Ion Current (TIC) (negative) of Jinshui-Huanxian granules (JHG)

Table S1. Main pharmacokinetic parameters of the control groups after intragastric administration of JHF. All data are presented as the mean ± SD. （$\overline{X}$±S, n=6）

Compounds	C_{max} (ng/mL)	$AUC_{(0-t)}$ (ng/mL·h)	$AUC_{(0-\infty)}$ (ng/mL·h)	T_{max} (h)	$t_{1/2}$ (h)	Clearances (L/h/kg)	$MRT_{(0-t)}$ (h)	$MRT_{(0-\infty)}$ (h)	Vss (L)
**Paeonol**	6.6±1.48<br>11.68±2.03<br>15.72±1.66	42.41±8.94<br>48.6±10.06<br>105.8±6.95	55.56±12.98<br>59.21±14.79<br>130.64±20.95	0.75±0<br>0.75±0<br>2±0	13.23±1.77<br>11.12±1.41<br>9.02±3.68	96.12±25.03<br>160.75±36.26<br>141.44±23.61	7.84±0.03<br>6.91±0.23<br>7.77±0.37	16.14±1.86<br>12.71±1.67<br>13.34±3.52	1528.44±295.01<br>2003.58±215.42<br>1831.33±158.35
**Nobiletin**	2.51±0.77<br>3.02±0.58<br>4.46±1.06	15.57±4.99<br>14.36±4.14<br>17.94±4.56	21.14±6.95<br>22.28±6.8<br>25.92±9.57	0.75±0<br>0.75±0<br>0.75±0	13.49±1.64<br>18.68±2.51<br>16.19±7.02	16.29±3.64<br>27.99±8.59<br>48.34±15.08	8.63±0.2<br>8.19±0.19<br>7.37±0.43	17.79±2.23<br>23.32±3.85<br>19.53±7.12	287.56±59.62<br>642.56±174.23<br>882.05±234.11
**Peimisine**	1.49±0.16<br>2.08±0.54<br>2.35±0.53	12.45±2.56<br>17.84±4.48<br>34.92±8.14	19.5±5.73<br>26.87±7.93<br>48.89±9.74	4±0<br>4±0<br>8±0	16.51±2.09<br>15.16±1.25<br>11.37±0.71	0.15±0.04<br>0.19±0.05<br>0.2±0.03	9.16±0.22<br>9.14±0.03<br>11.34±0.11	22.84±3.24<br>21.33±1.78<br>19.75±0.92	3.26±0.57<br>3.98±0.82<br>3.96±0.82
**Peiminine**	2.91±0.9<br>4.22±1.27<br>5.31±1.61	29.08±8.48<br>38.65±13.2<br>41.98±11.72	31.89±9.55<br>43.88±16.35<br>46.14±13.69	4.67±1.15<br>4±0<br>3±0	6.06±0.22<br>7.97±0.84<br>6.82±0.57	2.77±0.84<br>3.66±1.12<br>6.72±1.81	8.01±0.09<br>8.23±0.25<br>7.84±0.1	10.17±0.32<br>11.34±1.03<br>10.12±0.42	28.03±8.27<br>40.68±9.67<br>67.52±15.96
**Peimine**	0.57±0.22<br>1.49±0.26<br>2.53±0.56	7.38±2.13<br>14.58±1.9<br>26.58±4.11	24.73±5.52<br>17.65±2.24<br>29.93±4.92	4±0<br>3.33±0.58<br>2.67±0.58	52.06±30.73<br>8.02±0.39<br>7.23±0.62	1.86±0.47<br>4.53±0.57<br>5.32±0.73	11.1±0.12<br>8.8±0.32<br>8.43±0.11	75.32±41.77<br>13.46±0.58<br>11.32±0.63	131.39±53.45<br>61.2±9.95<br>60.06±7.12
**Cynaroside**	43.95±5.61<br>57.73±5.09<br>79.54±3.5	290.14±24.36<br>402±39.16<br>438.04±27.59	312.14±25.99<br>433.12±43.54<br>471.52±31.33	6±0<br>6±0<br>6±0	5.75±0.55<br>5.75±0.11<br>5.1±0.13	0.06±0.01<br>0.08±0.01<br>0.15±0.01	7.83±0.02<br>8.56±0.09<br>7.51±0.01	9.56±0.13<br>10.26±0.15<br>9.2±0.11	0.6±0.08<br>0.83±0.06<br>1.35±0.09

**Hesperidin**	3.21±0.67	22.12±3.51	25.8±4.55	2±0	7.7±0.63	195.87±17.16	7.52±0.11	11.4±0.44	2228.19±131.09
	9.01±1.03	56.74±8.01	60.9±8.78	3±0	5.14±0.25	148.43±20.1	6.2±0.18	7.91±0.43	1170.37±124.18
	10.27±1.47	70.01±8.49	96.38±13.43	3±0	17.64±6.08	186.2±31.33	7.55±0.24	19.29±6.7	3498.95±860.43
**Ginsenoside Rb_1**	8.54±0.92	92.72±6.92	140.14±22.61	5.33±1.15	14.83±4.95	5.5±1.16	9.74±0.53	21.93±6	115.88±11.46
	36.65±6.14	193.52±40.15	286.62±70.82	1.5±0	15.9±12.05	4.95±1.57	8.09±0.52	21.73±13.11	98.14±39.67
	73.68±6.13	516.19±26.29	624.04±99.95	1.5±0	9.1±4.28	4.34±0.71	7.71±0.52	12.85±4.23	53.81±7.92
**Ginsenoside Rb_2**	18.54±2.52	105.98±15.54	126.15±24.65	0.5±0	8.5±0.48	5.69±1.15	7.25±1.01	11.7±2.17	64.99±2.67
	29.35±2.39	119.72±13.55	162.39±48.14	0.5±0	14.41±6.88	8.08±1.98	7.45±0.38	17.16±7.89	128.29±24.51
	48.52±5.05	175.37±25.35	220.83±43.54	0.5±0	11.5±1.62	11.47±2.05	7.12±0.36	13.89±2.08	156.49±6.77
**Ginsenoside Rc**	5.06±0.74	65.34±9.13	106.24±46.97	4.67±1.15	16.43±9.45	7.42±2.84	10.12±0.36	24.24±12.3	157.55±20.92
	13.36±1.28	125.93±11.87	135.62±15.56	3.33±0.58	5.79±0.58	9.33±1.27	7.68±0.27	9.42±0.82	87.15±5.34
	21.97±2.21	180.61±19.79	210.55±24.72	2.67±0.58	7.84±0.67	11.86±1.23	7.96±0.24	11.84±0.36	140.17±11.84
**Icariin**	3.48±0.65	16.03±2.39	27.34±9.45	0.083±0	21.46±8.07	38.71±11.38	8.53±0.57	27.35±10.67	978.36±136.13
	5.11±0.56	21.24±2.58	27.51±3.65	0.083±0	10.68±2.11	64.85±7.46	7.8±0.22	14.99±0.93	971.6±122.73
	7.68±1.54	26.41±6.87	37.12±9.77	0.083±0	12.26±2.42	98.06±22.27	8.33±0.4	17.95±0.96	1766.12±449.66
**Catalpol**	4.74±0.33	36±5	40.71±7.32	3.33±0.58	8.07±1.5	0.26±0.06	7.56±0.41	10.73±1.77	2.67±0.25
	7.28±1.13	47.29±6.22	53.74±6.51	1.5±	7.72±0.04	0.34±0.04	7.64±0.12	10.96±0.16	3.72±0.45
	9.2±0.59	34.64±3	51.06±7.12	0.92±0.14	17.32±4.81	0.71±0.11	7.64±0.29	20.98±4.11	14.68±2.04
**Rutin**	10.78±2.18	21.44±8.06	44.34±32.27	0.083±0	29.9±16.87	0.46±0.23	7.33±0.65	36.15±22.73	13.09±2.88
	14.72±1.48	27.94±5.14	34.53±7.86	0.083±0	12.12±0.95	0.83±0.16	5.85±0.43	12.48±1.51	10.15±0.94
	19.23±2.32	30.72±5.92	55.86±21.64	0.083±0	31.15±15.78	1.12±0.53	6.35±0.43	34.55±19.24	32.16±11.41

**Apigenin-7-glucoside**	1.99±0.17<br>3.22±0.44<br>3.88±0.42	20.23±1.99<br>22.23±2.68<br>36.55±5.3	29.09±6.32<br>28.91±6.86<br>46.51±7.55	1.83±0.2<br>1.5±0<br>1.67±0.29	15.36±4.12<br>10.99±5.32<br>9.62±0.54	0.2±0.04<br>0.36±0.08<br>0.44±0.07	8.84±0.05<br>7.89±0.21<br>8.8±0.07	20.09±4.89<br>15.27±4.77<br>15±0.61	3.94±0.41<br>5.33±0.41<br>6.6±0.86
**Wedelolactone**	18.55±3.98<br>27.83±2.35<br>35.4±2.29	109.53±24.74<br>242.14±32.4<br>220.65±17.2	139.11±45.63<br>275.6±35.59<br>262.29±13.51	2±0<br>3.67±0.58<br>1.83±0.29	10.72±3.69<br>8.01±1.33<br>8.2±0.63	0.003±0.001<br>0.003±0<br>0.006±0	7.52±0.34<br>8.23±0.18<br>7.5±0.21	14.01±3.66<br>11.59±1.32<br>12.01±1.32	0.04±0<br>0.03±0.01<br>0.07±0.01
**Isoacteoside**	5.67±1<br>9.31±1.05<br>18.44±1.98	32.21±4.75<br>54.91±7.53<br>96.4±18.12	35.79±5.5<br>61.54±8.17<br>104.4±22.39	1±0<br>2±0<br>3±0	6.86±0.48<br>8.24±4.54<br>6.1±0.77	0.91±0.18<br>0.94±0.1<br>1.12±0.23	7.15±0.14<br>7.03±0.29<br>6.64±0.33	9.81±0.84<br>10.43±4.06<br>8.58±0.98	8.97±2.22<br>9.67±3.39<br>9.42±1.03

Table S2. Main pharmacokinetic parameters of the PF model group after intragastric administration of JHF. All data are presented as the mean ± SD. （X̅±S, n=6）

Compounds	C_{max} (ng/mL)	$AUC_{(0-t)}$ (ng/mL·h)	$AUC_{(0-\infty)}$ (ng/mL·h)	T_{max} (h)	$t_{1/2}$ (h)	Clearances (L/h/kg)	$MRT_{(0-t)}$ (h)	$MRT_{(0-\infty)}$ (h)	V_{ss} (L)
**Paeonol**	2.79±0.6*<br>3.16±0.22**<br>3.63±0.33**	21.82±2.86*<br>24.54±2.15*<br>34.97±1.86**	41.77±12.11<br>41.58±9.78<br>83.36±34.32	0.5±0<br>0.75±0<br>3±0	25.5±8.84<br>19.16±6.63<br>36.4±25.65	93.8±25.66<br>316.18±67.77*<br>282.84±90.76	9.14±0.1**<br>9.7±0.37**<br>9.41±0.4**	33.81±11.44<br>26.89±8.95<br>49.6±33.28	2980.29±449.33**<br>8115.77±895.52**<br>12017.41±3866.6*
**Nobiletin**	2.97±0.67<br>3.37±0.53<br>3.57±0.3	21.64±3.48<br>20.03±2.62<br>15.48±2.99	33.4±4.41<br>43.11±11.09<br>17.96±3.39	0.75±0<br>0.75±0<br>0.75±0	17.64±1.82*<br>31.46±13.26<br>9.59±3.39	7.16±0.71*<br>19.65±5.02<br>78.42±15.85	8.86±0.2<br>9.13±0.25**<br>6.66±0.35	23.25±2.12*<br>41.81±18.37<br>11.05±1.44	166.86±26.65*<br>769.75±168.2<br>864±195.9
**Peimisine**	2.05±0.49<br>2.51±0.55<br>2.76±0.48	12.31±2.23<br>23.44±5.01<br>29.71±5.82	14.62±2.48<br>26.76±5.8<br>33.14±6.82	1.5±0<br>3.67±0.58<br>8±0	7.2±0.28**<br>8.25±0.94**<br>5.96±0.23**	0.14±0.02<br>0.26±0.05<br>0.35±0.06*	7.56±0.17**<br>7.92±0.2**<br>9.84±0.24**	11.83±0.51**<br>11.39±1.05**<br>12.17±0.39**	1.62±0.32*<br>2.91±0.52<br>4.24±0.61

**Peiminine**	2.9±0.47<br>6.1±1.14<br>7.52±1.33	22.85±2.61<br>46.56±8.45<br>45.99±7.27	39.76±7.22<br>53.38±10.3<br>60.7±7.68	3±0<br>2±0<br>2±0	23.2±5.2**<br>6.9±0.28<br>13.32±1.69**	1.54±0.23<br>3.94±0.72<br>5.66±0.74	8.41±0.09**<br>7.7±0.15*<br>8.23±0.1**	29.31±6.36**<br>11.03±0.46<br>16.82±1.78**	44.32±5.6*<br>43.3±6.88<br>95.87±20.6
**Peimine**	0.58±0.28<br>1.72±0.37<br>2.19±0.54	6.96±3.07<br>20.41±3.87<br>16.76±3.5*	10.76±3.94*<br>37.1±9.76*<br>19.8±4.16	2±0<br>2.33±0.58<br>2±0	14.3±1.49<br>21.76±6.99*<br>8.17±1.23	3.22±0.99<br>3.11±0.86<br>9.64±1.83*	9.84±0.23**<br>9.82±0.12**<br>7.82±0.18**	22.57±2.38<br>30.39±8.97*<br>12.12±1.15	74.22±28.56<br>91.13±22.81<br>116.13±19.39**
**Cynaroside**	46.2±2.06<br>83.26±7.88**<br>134.6±8.44**	423.85±24.91**<br>560.68±41.87**<br>958.86±41.44**	476.73±29.92**<br>614.53±46.51**<br>1036.81±55.15**	3±0<br>6±0<br>6±0	7.21±0.57*<br>5.37±0.13*<br>5.13±0.72	0.03±0**<br>0.08±0.01<br>0.08±0.01**	8.78±0.26**<br>9.11±0.12**<br>7.94±0.51	11.62±0.81*<br>11.09±0.19**<br>9.7±1.19	0.34±0.02**<br>0.88±0.08<br>0.76±0.08**
**Hesperidin**	5.05±1.07<br>6.94±1.14<br>9.73±1.58	26.67±4.06<br>38.5±5.98*<br>36.83±5.42**	40.84±14.26<br>48.11±7.84<br>53.46±11.2*	0.5±0<br>0.58±0.14**<br>0.75±0	15.89±9.61<br>9.55±3.8<br>17.76±2.85	94.98±28.23**<br>261.62±41.62*<br>401.29±71.2**	8.25±0.12**<br>7.56±0.32**<br>7.59±0.12	21.69±9.82<br>13.74±3.36*<br>20.55±3.29	1878.92±191.23<br>3564.26±885.36**<br>8093.51±500.52**
**Ginsenoside Rb_1**	8.58±1.21<br>12.41±1.04**<br>29.18±2.09**	64.71±8.4*<br>132.19±16.63<br>275.87±11.73**	93.5±5.54*<br>177.59±32.82<br>451.38±35.94*	2±0**<br>3±0<br>3±0	17.19±2.77<br>11.71±1.42<br>18.14±1.31*	5.85±0.49<br>10.76±2.16*<br>7.01±0.44**	8.06±0.06**<br>9.41±0.22*<br>9.43±0.01**	20.8±3.1<br>17.35±2.14<br>25.24±1.65**	122.64±27.46<br>183.67±15.59*<br>176.57±2.42**
**Ginsenoside Rb_2**	7.13±0.66**<br>10.68±0.81**<br>13.53±1.31**	39.02±5.58**<br>51.23±7**<br>102.3±15.13*	44.68±9.34**<br>63.18±8.7*<br>113.4±19.38*	1.5±0<br>0.75±0<br>2±0	8.82±3.63<br>11.16±2.36<br>6.94±0.93*	11.62±2.13*<br>27.69±3**<br>26.4±5.05**	6.35±0.23<br>7.54±0.02<br>7.45±0.46	10.16±2.62<br>13.77±2.04<br>9.99±1.21*	114.37±8.23**<br>380.31±62.68**<br>259.84±18.48**
**Ginsenoside Rc**	9.7±0.81**<br>7.45±1.72**<br>12.67±1.2**	60.82±4.16<br>51.37±6.66**<br>117.75±15.87*	65.09±4.04<br>59.74±9.14**<br>134.85±21.57*	2.67±0.58<br>0.5±0**<br>6±0**	5.95±0.44<br>8.98±0.81**<br>6.95±1.23	7.81±0.52<br>29.46±4.2**<br>22.15±3.72*	6.73±0.45**<br>7.97±0.54<br>8.95±0.43*	8.42±1<br>11.98±1.19*<br>12.09±1.31	65.52±5.84**<br>349.52±13.66**<br>264.75±16.5**

**Icariin**	5.03±1.67<br>8.94±2.26*<br>33.76±2.43**	17.33±4.3<br>25.98±7.94<br>41.42±8	25.58±8.7<br>39.82±18.43<br>50.71±12.55	0.083±0<br>0.083±0<br>0.083±0	15.46±3.04<br>18.58±8.45<br>13.14±2.84	30.05±9.19<br>70.43±30.22<br>85.16±21.02	8.67±0.19<br>7.64±0.44<br>5.24±0.29**	20.49±3.84<br>22.18±9.82<br>12.01±2.57*	593.51±107.45*<br>1375.51±378.99<br>986.8±70.97*
**Catalpol**	2.41±0.19**<br>2.93±0.39**<br>4.92±0.5**	22.81±1.65*<br>21.55±3.64**<br>40.89±5.78	26.81±2.42*<br>28.67±7.59*<br>48.38±9.34	3±0<br>3±0<br>3±0**	8.26±0.23<br>12.24±3.62<br>9.37±1.69	0.27±0.02<br>0.91±0.23<br>0.89±0.13	8.29±0.08*<br>8.16±0.24*<br>7.78±0.24	12.38±0.36<br>16.31±3.81<br>12.3±1.88*	3.39±0.17*<br>14.32±0.95**<br>10.82±0.73*
**Rutin**	16.59±2.86*<br>25.8±3.39**<br>37.05±3.02**	28.48±5.62<br>49.09±8.67*<br>66.33±19.05*	46.79±20.43<br>71.34±23.58<br>88.67±20.05	0.083±0<br>0.083±0<br>0.083±0	23.27±10.82<br>16.2±5.74<br>15.47±8.31	0.27±0.02<br>0.91±0.23*<br>0.89±0.13	6.89±0.47<br>7.85±0.45**<br>6.74±0.54	26.14±13.74<br>19.85±7.03<br>17.57±8.16**	6.07±1.1*<br>10.63±1.35<br>13.32±6.2
**Apigenin-7-glucoside**	0.89±0.09**<br>1.12±0.21**<br>1.29±0.19**	10.33±2.48**<br>10.37±2.06**<br>13.53±3.26**	37±23.83<br>35.08±20.21<br>35.78±10.37	0.75±0**<br>0.75±0<br>1±0*	51.09±30.25<br>50.06±35.82<br>36.79±10.35*	0.15±0.1<br>0.5±0.28<br>0.71±0.19	10.28±0.35**<br>10.35±0.33**<br>10.46±0.19**	72.46±41.27<br>71.08±50.33<br>52±14.25*	8.27±1.79*<br>28.13±6.93**<br>35.51±8.06**
**Wedelolactone**	28.94±4.76*<br>33.45±4.8<br>57.72±4.16**	123.16±20.76<br>208.21±30.44<br>292.92±30.82*	147.59±26.83<br>239.66±37.23<br>321.22±41.64	0.75±0<br>1.5±0**<br>2±0	9.87±3.09<br>8.45±1.09<br>7.16±1.58	0.002±0<br>0.005±0.001*<br>0.006±0.001	7.09±0.38<br>7.17±0.05**<br>6.21±0.47*	12.32±3.09<br>10.97±0.54<br>8.62±1.19*	0.03±0.01**<br>0.05±0.01*<br>0.05±0*
**Isoacteoside**	5.09±0.84<br>11.05±1.79<br>14.56±1.45	51.74±8.76*<br>102.39±22.45*<br>152.04±19.45*	122.01±76.74<br>129.24±35.86*<br>165.86±24.94*	3.33±0.58**<br>6±0<br>8±0	35.69±34.63<br>9.25±2.16<br>5.35±0.65	0.24±0.12**<br>0.64±0.16<br>0.82±0.1	9.32±0.41**<br>8.95±0.5**<br>9.78±0.25**	48.85±47.3<br>14.71±2.44<br>11.57±0.81*	8.18±3.1<br>9.2±1.33<br>9.42±0.8

* indicates $p < 0.05$ compared with the control group of the same dose, statistically difference;
** indicates $p < 0.01$, statistically high difference
